# Establishment of CD1b-restricted immunity to lipid antigens in the pulmonary response to *Mycobacterium tuberculosis* infection

**DOI:** 10.1128/iai.00380-24

**Published:** 2024-11-04

**Authors:** Macallister C. Harris, Hadley E. Gary, Sarah K. Cooper, David F. Ackart, James E. DiLisio, Randall J. Basaraba, Tan-Yun Cheng, Ildiko van Rhijn, D. Branch Moody, Brendan K. Podell

**Affiliations:** 1 Mycobacteria Research Laboratories, Department of Microbiology, Immunology and Pathology, Colorado State University, Fort Collins, Colorado, USA; 2 Brigham and Women’s Hospital, Division of Rheumatology, Inflammation and Immunity, Harvard Medical School, Boston, Massachusetts, USA; Rutgers-New Jersey Medical School, Newark, New Jersey, USA

**Keywords:** CD1, CD1b, *Mycobacterium*, *tuberculosis*, lipid antigen, guinea pig, pulmonary immunity

## Abstract

CD1 is an antigen-presenting glycoprotein homologous to MHC I; however, CD1 proteins present lipid rather than peptide antigens. CD1 proteins are well established to present lipid antigens of *Mycobacterium tuberculosis* (Mtb) to T cells, but understanding the role of CD1-restricted immunity *in vivo* in response to Mtb infection has been limited by the availability of animal models naturally expressing the CD1 proteins implicated in human response: CD1a, CD1b, and CD1c. Guinea pigs, in contrast to other rodent models, express four CD1b orthologs, and here we utilize the guinea pig to establish the kinetics of gene and protein expression of CD1b orthologs, as well as the Mtb lipid-antigen and CD1b-restricted immune response at the tissue level over the course of Mtb infection. Our results indicate transient upregulation of CD1b expression during the effector phase of adaptive immunity that wanes with disease chronicity. Gene expression indicates that the upregulation of CD1b is the result of transcriptional induction across all CD1b orthologs. We show high CD1b3 expression on B cells, and identify CD1b3 as the predominant CD1b ortholog in pulmonary granuloma lesions. We identify *ex vivo* cytotoxic activity directed against CD1b that parallels the kinetic changes in CD1b expression in Mtb-infected lungs and spleen. This study confirms that CD1b expression is modulated by Mtb infection in lung and spleen, leading to pulmonary and extrapulmonary CD1b-restricted immunity as a component of the antigen-specific response to Mtb infection.

## INTRODUCTION

Expanding the perspective that T cells recognize MHC-peptide complexes, CD1-lipid complexes activate invariant natural killer T cells that specifically respond to CD1d, while other T cells recognize lipid antigens presented by CD1a, CD1b, and CD1c (1, 2). CD1 glycoproteins have homologous structuring to MHC I antigen-presenting molecules but bind and present a variety of lipid antigens (3, 4). In humans, four CD1 antigen-presenting molecules are divided into two functional groups; group 1 is composed of CD1a, CD1b, and CD1c, and group 2 is composed of CD1d (5–7). Group 2 CD1 complexes are constitutively expressed on many antigen-presenting cell (APC) types, including macrophages, B cells, and myeloid dendritic cells, where they present sphingoglycolipids for expansion of invariant natural killer T cells (8, 9). By contrast, Group 1 CD1 proteins have an inducible expression on myeloid dendritic cells, where they load endogenous and exogenous lipids, such as those derived from *Mycobacterium tuberculosis* (Mtb), leading to an expansion of a variety of functionally nonpolymorphic CD1-restricted αβ and γδ T cells (10–13).

Owing to the lipid-rich composition of Mtb, multiple studies have investigated the potential role of CD1-restricted immunity in tuberculosis (TB). Studies examining the peripheral blood of Mtb-infected human subjects found increased expression of CD1 on myeloid cells and the corresponding presence of CD1-restricted T cells (14–17). Other studies have taken additional strides in the isolation of Mtb-lipid-specific T cells *via* detection with lipid-loaded CD1 tetramers (18, 19). Though these studies lay a foundational understanding of CD1-restricted immunity during Mtb infection by identifying polyclonal T-cell responses *ex vivo*, many gaps remain: whether CD1-restricted immunity contributes as a component of innate or adaptive branches of immunity *in vivo*, whether CD1 expression is modulated by Mtb infection *in vivo*, and whether CD1 plays a functional role in disease pathogenesis and granuloma formation in the tissue response to infection (6, 7, 16).

One major limitation to further understanding of CD1 lipid-restricted immunity in response to Mtb is the lack of a translatable animal model with naturally occurring group 1 CD1 expression. Experimentally tractable mouse models naturally express only CD1d. However, guinea pigs have a similar CD1 complex repertoire to humans, containing four orthologs of the group 1 CD1b protein and three orthologs of CD1c, as well as CD1d (20, 21). Previous studies have characterized this CD1 repertoire *in vivo* and *ex vivo* as well as in the face of administered lipids isolated from cultured Mtb and BCG vaccination (20, 22, 23). However, there is no understanding of how Mtb infection impacts CD1 expression and the development of CD1-restricted T-cell immunity over the course of TB disease in infected tissue. In this study, we utilized the guinea pig model of pulmonary TB and reagents specific to CD1b orthologs to kinetically measure CD1b-restricted immunity at multiple stages of TB disease within the lung and spleen. Overall, this work demonstrates that CD1-restricted immunity actively responds to Mtb, as manifested by CD1b-mediated cytolytic responses to mycobacterial lipid antigens, indicating a pathogen-specific *in vivo* response to infection. Furthermore, this study establishes the ability to assess CD1b-driven immunity at the protein and molecular level within infected tissue utilizing a translatable small animal model.

## MATERIAL AND METHODS

### Animals

Two separate animal studies were performed to kinetically monitor CD1b expression in blood and tissue over the course of Mtb infection. Female outbred Dunkin-Hartley guinea pigs weighing 250–300 g were obtained from either Charles River Laboratories (Wilmington, MA) or Elm Hill Labs (Chelmsford, MA). In the first study, guinea pigs infected with Mtb were evaluated at 14, 30, and 60 days post-infection (DPI) (*n* = 6 per endpoint). In the second study, Mtb-infected guinea pigs at 14, 30, and 60 DPI (*n* = 5 per endpoint) were compared to uninfected, naïve guinea pigs (*n* = 3) at each necropsy endpoint to compare the impact of infection over baseline CD1b expression. Tissues and cells from inbred strain 13 guinea pigs, a colony maintained at Colorado State University, were utilized for assay development, including cytotoxicity, CD1 expression *via* flow cytometric analysis, and validation of antibodies for immunohistochemistry on fresh frozen tissue. All animals were monitored *via* the Colorado State University (CSU) Lab Animal Resources staff and veterinarians and performed in accordance with protocols approved by the Institutional Animal Care and Use Committee protocol number 1401.

### CD1-specific reagents

To separately measure responses to the four CD1b orthologs in guinea pigs, we used 104C1 guinea pig fibroblast cell lines transfected with CD1b1, CD1b2, CD1b3, and CD1b4 (20). Specific CD1b ortholog-expressing cell lines were compared to a mock transfectant containing only the empty pcDNA3.1 vector. Synthetic lipid antigens, glucose monomycolate and mycolic acid (24), were used to eliminate the possibility of co-purifying peptide antigens in preparations, which was essential to avoid MHC-restricted T-cell responses. These lipid antigens were chosen as known CD1b ligands with demonstrated antigen-specific response in previous studies with antigen-specific IFNγ production or antigen-specific CD1b tetramer-based detection of cells in human peripheral blood (24–26). CD1-transfected 104C1 cells (CD1b1-4 and CD1c1-3) and anti-guinea pig CD1 mouse monoclonal antibodies allowed analysis of ortholog specificity (Fig. S1 and S2; Table S1) (20).

### Infection and euthanasia

Low-dose aerosol exposure of guinea pigs to Mtb was performed using the Madison chamber aerosol generation device (College of Engineering Shops, University of Wisconsin, Madison, WI) calibrated to deliver approximately 20–50 bacilli of the H37Rv strain of Mtb isolated during log-phase growth in Proskauer-Beck media. A second study used the Glas-Col aerosol chamber (Terre Haute, IN) to deliver a low-dose exposure of <50 bacilli of the Erdman strain of Mtb. On pre-determined endpoints, 14, 30, and 60 days post-infection, guinea pig groups were euthanized *via* an initial induction of an anesthetic state *via* intramuscular injection of 30 mg of ketamine and 8 mg of xylazine in combination, followed by an intraperitoneal injection of 3 mL/animal of 390 mg/mL sodium pentobarbital.

### Necropsy and tissue processing

Animal tissues and cells were harvested, which included airway-accessible macrophages and other leukocytes by bronchoalveolar lavage, peripheral blood mononuclear cells (PBMCs) from anticoagulated peripheral blood, and cell suspensions derived from lung and splenic parenchyma, as previously reported (27, 28). In brief, the right cranial and caudal bronchi were clamped and lung lobes were removed and processed for either RNA preservation in RNAlater solution (Thermofisher, Waltham, MA), OCT-embedding for fresh-frozen immunohistochemistry (IHC) (Sakura, Torrance, CA), or for bacterial enumeration by colony forming units (CFU). Airway-accessible leukocytes were collected *via* postmortem bronchoalveolar lavage utilizing an 18-gauge catheter and flushing with 25 mL of HBSS containing 0.01 µM EDTA solution in five, 5 mL, intervals. Following bronchoalveolar lavage, the lung was perfused with 5 µg/mL collagenase (Sigma-Aldrich, cat#: C9263) and incubated for 30 minutes in a 37°C water bath. The digested lung parenchyma and collected spleen were individually processed through 40 µm cell strainers. Once strained, all samples were subjected to hypotonic solution for 20 to 45 seconds for lysis of erythrocytes. To increase overall cell viability for downstream assays, all tissue-derived cell suspensions were subjected to magnetic bead negative selection using biotinylated annexin V and streptavidin-conjugated magnetic nanobeads. Cells were placed in 4 mL of 1× annexin binding buffer (Biolegend, cat#: 422201) with biotinylated annexin V (Biolegend, Cat # 640904) and incubated for 30 minutes then centrifuged at 500 × *g* for 5 minutes, washed with binding buffer, centrifuged again, and resuspended in 500 µL of binding buffer. Thirty microliters of streptavidin nanobeads (Biolegend, cat#: 480016) was added and incubated for 20 minutes with intermittent vortexing. Additional binding buffer was added to bring solutions to 4 mL and the cell suspension was placed on a magnetic rack for 10 minutes to allow for magnetic binding of nanobeads. Finally, unbound cells were collected with a serologic pipette. Collected cells were washed in Hank’s balanced salt solution and then viability and cell count were assessed *via* a hemocytometer and trypan blue exclusion. Splenic and lung suspension cells were used for the analysis of CD1b expression by flow cytometry and the remaining cells were plated in RPMI media containing 10% fetal bovine serum and an antimicrobial cocktail and then incubated overnight at 37°C with 5% CO_2_ to allow for separation of adherent cells (histiocytic/dendritic in origin) and non-adherent cells (lymphocytes). PBMCs were collected *via* a right ventricular intracardiac blood draw directly following euthanasia, diluted 1:2 in HBSS, and separated on a density gradient using lympholyte mammal media (Cedarlane, cat#: CL5110) per the manufacturer’s instructions.

### Intradermal *M. tuberculosis* antigen challenge

In the second study, 48 hours prior to euthanasia, guinea pigs received an intradermal hypersensitivity challenge to Mtb lipid antigens, Mtb protein antigen in the form of purified protein derivative (PPD), or sterile PBS. Each guinea pig received seven intradermal injections: (i) mycolic acid liposome, (ii) glucose monomycolate liposome, (iii) mycolic acid in PBS, (iv) glucose monomycolate in PBS, (v) unloaded liposome, (vi)PPD (Tuberculin PPD Bovine, USDA Veterinary Services), or (vii) sterile PBS. Liposomes were made using distearoylphosphatidylcholine (DSPC) (Avanti lipids, cat#: 850365P) and cholesterol (Avanti lipids, cat#: 700100P) lipids with or without synthetic Mtb lipid (molar ratio 7:2:1, respectively) *via* a lipid extruder (Avanti Lipids, Alabaster, AL). For liposomal formulation, DSPC and cholesterol were added to a pre-rinsed glass container, dissolved in a solution of 10:1 chloroform and methanol, and solubilized mycolic acid or glucose monomycolate was added. The lipid solution was completely evaporated utilizing a nitrogen gas bath. Dried lipids were then suspended in PBS prewarmed to 68°C. The solution was then vortexed and incubated in a water bath at 68°C for 40 minutes, with vortexing every 10 minutes, to allow for liposome formation. During the incubation, the liposome extruder apparatus (Avanti lipids, Alabaster, AL) was assembled to company specifications and warmed to 70°C. After incubation, the liposome solution was passed through the extruding system 7–8 times to allow for the formation of uniform unilamellar liposomes at approximately 150 µm in diameter. The liposomal size was confirmed with a Nanosight instrument and capacity for macrophage phagocytosis was confirmed using guinea pig bone marrow-derived macrophages in *in vitro* culture (Fig. S2). PBS lipid suspensions were sonicated using a Misonix ultrasonic liquid processor at 80 A for 4 minutes on a 20-second-on, 20-second-off cycle. All lipid injection sites received 4 µg of lipid whether in liposomal formulation or free lipid in PBS. PPD was administered at 2 µg per site. Guinea pigs were ventrally shaved from the point of the caudal xiphoid to the cranial aspect of the pubis. Injection sites were labeled and administered *via* intradermal injection using an insulin syringe at 50 µL of sample per site. All sites were measured at 24 hours and 48 hours after the initial injection using calipers to measure cranial to caudal and lateral diameters.

### Kinetic CD1 expression

CD1b expression was measured by flow cytometry on cell suspensions from lung and spleen, bronchoalveolar lavage, and PBMCs. In brief, published methods (27, 28) used 1.5 × 10^6^ cells that were blocked with 2.24 µg/mL of guinea pig and rabbit immunoglobulin (Jackson Immunoresearch, Cat#: 011-000-003 and 006-000-002) in FACS buffer (PBS containing 1% FBS and 0.01% sodium azide) or Fc-receptor block (Innovex, Cat#: 50-486-808). Cells were labeled with an antibody panel targeting CD1b expression (Table S2). The viability of cells was assessed *via* Zombie Yellow amine-reactive permeability dye (BioLegend, Cat# 423103). Data were acquired using a spectral unmixing Cytek Aurora flow cytometer (Cytek, Fremont, CA) and analyzed *via* Flowjo software version 10.8 (Flowjo, Ashland, OR) using a minimum of 100,000 events (Fig. S3). CD1b expression was inferred by comparing the mean fluorescence intensity (MFI) values for lung and spleen cells from each infected guinea pig to naïve guinea pigs after applying the same gating strategy. Further evaluation of CD1b expression on leukocytes with a small scatter profile was performed on naïve uninfected guinea pig spleen cells. Spleen and lung cell suspensions were labeled with anti-guinea pig CD45-FITC, anti-guinea pig CD1b-PE, anti-guinea pig IgG-APC, and an anti-guinea pig B-cell antibody (Bio-Rad antibodies, clone MsGp10) with specificity consistent with CD19 and conjugated to AlexaFluor 594. Gating was performed to evaluate the expression of surface IgG and CD19 on CD1b-expressing leukocytes using Flowjo software.

### CD1 cytotoxicity

Building on prior evidence for cytotoxicity with lipid antigens (29, 30), we measured the cytotoxic activity of CD1b-restricted immunity over the course of Mtb infection. CD1b fibroblast transfectants served as target cells and were incubated with non-adherent cells isolated from the lung or spleen of each animal. Detection of cytotoxicity, assayed as membrane permeability, was performed using a published method (31). In brief, 1 × 10^7^ CD1b1- and CD1b3-transfected cells were collected and incubated with 0.5 µM Cell Trace Violet (Thermofisher, C34557) for 15 minutes in RPMI media. After quenching with 10 mL of RPMI containing 10% FBS for 10 minutes, cells were washed twice and plated in a 96-well plate at 5 × 10^4^ per well. Free lipid solutions were prepared as described for the intradermal skin testing and resuspended in RPMI media using mock-loaded and unloaded controls for determination of CD1b-restriction and lipid-antigen specific cytotoxicity. Cells and lipids were incubated overnight to allow for lipid cellular trafficking and lipid presentation in the context of CD1b. These target cells were then incubated with non-adherent inflammatory cells derived from the lung and spleen from either naïve or Mtb-infected guinea pigs for 4 hours. Incubated cells were then stained for viability using Zombie Yellow dye, diluted at 1:500 in PBS, and then added at 100 µL per sample (BioLegend, Cat# 423103). Data were acquired using a Cytek Aurora spectral unmixing flow cytometer (Cytek, Fremont, CA) and analyzed *via* Flowjo software version 10.8 (Flowjo, Ashland, OR) using a minimum of 50,000 events with fibroblasts identified based on size and the presence of CellTrace Violet signal (Fig. S4). To address background cell death and background to signal ratios commonly impacting cell-mediated cytotoxicity assays (32–34), we incorporated two control conditions: mock-transfected fibroblasts, containing the empty vector with no CD1b construct, or CD1b1 and CD1b3 transfected fibroblasts without added lipid antigen, referred to as unloaded. Owing to the high cell number demand of this assay and the limited number of cells recovered from each animal, mock transfectant controls were performed as a single set for each infection endpoint at days 14, 30, and 60. Loaded and unloaded samples for each CD1b1 and CD1b3 lipid antigen restriction tested were prioritized and performed for each animal and tissue type at each endpoint of infection. A lipid antigen-specific cytotoxic effect was defined as a statistically significant increase in the proportion of non-viable target cells among antigen-loaded CD1b1/CD1b3-transfected targets above both the unloaded CD1b1/CD1b3-transfected targets and unloaded mock-transfected controls.

### Kinetic expression of specific CD1b orthologs by qRT-PCR relative gene expression

Kinetic gene expression of CD1b was separately measured with ortholog-specific primers (CD1b1-b4) designed using Geneious software (Auckland, NZ) *via* a 4-sequence alignment and selected for non-consensus primer sets, which were then synthesized by Integrated DNA Technologies (IDT) (Coralville, IA) (Table S3). Primer set specificity was evaluated *via* the use of synthetic gene segments from IDT for each CD1b sequence (Fig. S5), demonstrating specificity for each of the CD1b1-b4 orthologs without cross-reactive detection (Fig. S6). For RNA isolation, freshly isolated lung tissue was minced in RNALater (Sigma, cat#: R0901), incubated at 4°C for 24 hours, then frozen at −80°C until RNA extraction. Tissue was thawed at room temperature and approximately 50 mg of tissue homogenized in RLT buffer (Qiagen) containing β-mercaptoethanol using Lysing Matrix A tubes (VWR, cat# 75784-610) in a bead beater instrument at full speed for two cycles of 20 seconds (MP Biomedical, cat# C321001). Proteinase K was added to the homogenate and incubated at 55°C for 15 minutes, then RNA was extracted with the RNeasy mini kit (Qiagen, cat# 74106). Nucleic acid was eluted from the column, digested with DNase-I for 30 minutes at 37°C, then purified again using the RNeasy mini kit. Sample concentration was measured using a Nanodrop spectrophotometer and quality was analyzed on an Agilent TapeStation. Generation of cDNA was performed using the iScript cDNA synthesis kit (BioRad cat# 1708890), reverse transcribing 1 µg of RNA per reaction. 25 ng of cDNA template was added to an iScript Sybr Green master mix containing 1.5 mM MgCl_2_ and 0.1 µM of each forward and reverse primer. Relative gene expression was measured by normalizing log_2_-fold expression to reference genes HPRT and β-actin using the ΔΔCt method, as previously described (35, 36). Reference gene-normalized expression data were then plotted relative to the sample with the lowest expression of each CD1b-ortholog, which was represented by a naïve, uninfected control animal.

### Distribution of tissue CD1 expression by immunohistochemistry

Tissues from necropsy were embedded in OCT (Thermofisher, cat#: 23–730-571) and cryopreserved in a liquid nitrogen bath, stored at −80°C, and sectioned using a cryotome maintained at −16 to −18°C, and a 7-µm section adhered to charged glass slides. After sectioning, tissues were immediately placed in a 100% chilled methanol fixation for 45 minutes to fix the tissue and inactivate the Mtb organism. All DAB chromogenic and fluorescent immunohistochemistry protocols were completed on methanol-fixed tissue sections utilizing a Leica Bond RXm automated slide stainer (Tables S4 and S5). Fluorescent multiplex IHC was achieved using Opal fluorochromes 570 and 690 (Akoya Biosciences, cat# 570-FP1488001KT, 690-FP1495001KT, 690-FP1497001KT) to detect CD1-ortholog-specific expression and distribution across myeloid/histiocytic, based on morphology, and specific detection of B-cell subsets, based on Pax5 transcription factor expression (clone 24, CellMarque, cat# 312M). All tissues were blocked in 2.5% goat serum solution (Vector Labs, cat# S-1012-50) and primary antibodies were diluted in 2.5% goat serum. Primary antibodies were detected using a goat anti-mouse HRP secondary (Vector Labs, cat# MP-7452-50) and incubated with DAB substrate for chromogenic IHC or Opal fluorochromes for fluorescent IHC protocols. Quantification of immunohistochemistry was performed using Visiopharm (Hoersholm, Denmark). A nuclei-detect application was developed to discern between DAPI-stained nuclei and Pax5-positive nuclei as well as identify all cells within tissues, then further delineating cells based on CD1b expression.

### Quantification of tissue bacterial burden

Lung bacterial burden was quantified as previously described (37). In brief, the tissue homogenate was plated on 7H11 agar quad plates in five-fold serial dilutions, incubated for 8 weeks, and counted.

### Statistical analysis

All analyses, with the exception of the cytotoxicity assay, were performed utilizing Prism version 10.2.3 (GraphPad, San Diego, CA). Data distribution was evaluated *via* a Shapiro-Wilks test. Based on distribution, a two-way ANOVA test or Kruskal-Wallis test was performed to determine statistical significance for flow cytometric analysis, gene expression, and histologic quantifications. Statistical significance was set at *P* ≤ 0.05. For the cytotoxicity assay, analyses were performed using R Statistical Software (v4.2.1; R Core Team 2021). AUC was calculated based on the ratio of effector to target cells and normalized percent killing *via* the metrumrg R package (v5.57) (38). A one-way ANOVA with Tukey post hoc correction was used to determine the significance between groups with the rstatix R package (v0.7.0) (39).

## RESULTS

### CD1b expression during Mtb infection on lung and spleen leukocytes

**Fig 1:**
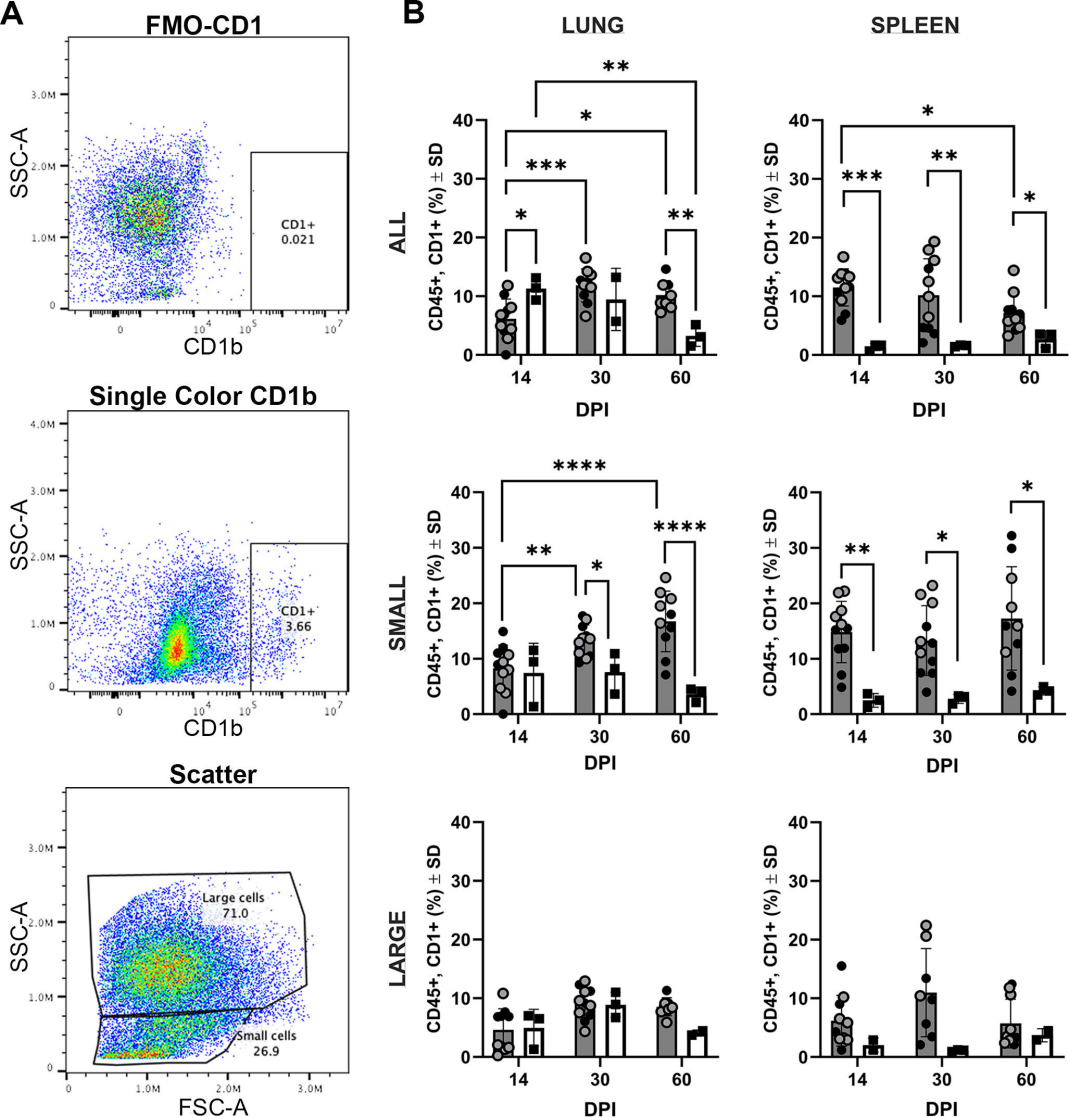
CD1b expression over the course of Mtb infection. Cells were gated for live, CD45+ cells and evaluated for overall CD1 expression using the anti-CD1b monoclonal antibody, 1B12. Open and closed circles represent two separate experimental sets of identical study designs. Naïve, time-matched controls were performed solely in one of two experiments. Circles represent Mtb-infected guinea pigs and squares represent uninfected guinea pigs. (**A**) Scatter plot gating of CD1-positive cells, demonstrating fluorescence-minus-one FMO gating control (top), gating of CD1b-expressing cells (middle), and gating strategy for isolating small vs large CD45+ leukocytes (bottom). (**B**) Proportion of cells expressing CD1b among lung- and spleen-derived cells as all CD45+ cells, delineated small leukocyte populations, and delineated large leukocyte populations. *P ≤ 0.05, **P ≤ 0.01, ***P ≤ 0.001, and ****P ≤ 0.0001. (**B**) Two-way ANOVA with Tukey post hoc correction for multiple comparisons. DPI (day post-infection).

*In vitro*, human antigen-presenting cells can induce CD1b in response to mycobacterial exposure, suggesting a natural response whereby CD1b can locally function near infection (15). To examine the kinetics *in vivo*, we evaluated the influence of Mtb infection on CD1b expression over the course of TB disease in two independent *in vivo* infection studies in the spleen and lung of Mtb-infected guinea pigs among viable CD45+ leukocytes. An increased frequency of CD1b+ cells was demonstrated among infected animals compared to time-matched naïve controls *via* flow cytometric analysis using the 1B12 antibody that recognizes multiple CD1b orthologs (Fig. 1A and B). Leukocytes derived from lung parenchyma had reduced (*P* < 0.05) surface CD1b expression at day 14 of infection (mean 6.2% ± 3.4%) compared to naïve uninfected controls (mean 11.3% ± 1.9%). The frequency of CD1b-expressing cells in lungs of infected animals was increased at day 30 of infection compared to day 14 (*P* < 0.001), where there were a mean of 11.8% ± 2.8% CD1b+ cells in the infected lung, compared to 9.4% ± 5.3% CD1b+ cells among naïve, uninfected guinea pigs. The frequency of CD1b expressing leukocytes remained similarly elevated at day 60 of infection with a mean of 10.2% ± 3.3% CD1b+ compared to day 14 of infection (*P =* 0.05) and was greater than naïve time-matched controls (*P* < 0.01). Splenic CD45+ leukocytes had increased surface CD1b expression at all Mtb infection endpoints evaluated (*P* < 0.05 to *P* < 0.001) with expression decreasing between day 14 and day 60 (*P* < 0.05).

Owing to the lack of an anti-guinea pig CD3 antibody for flow cytometry, we sought to further discriminate the CD45+ leukocyte populations expressing CD1b over the course of infection based on scatter parameters (Fig. 1B). Cells were gated by forward and side scatter to differentiate small and large leukocyte populations, previously shown to enrich for lymphocyte and myeloid populations, respectively (27, 28, 40). Large-cell leukocytes from either lung or spleen had no difference in expression of CD1b at days 14, 30, or 60 of Mtb infection or compared to time-matched naïve control guinea pigs. By contrast, lung-derived small-cell leukocytes had significant upregulation over the course of infection, increasing from 7.6% ± 3.6% CD1b+ at day 14 of infection to 16.7% ± 5.4% CD1b+ at day 60 of infection in the lung (*P* < 0.001). CD1b surface expression in the lung was greater in small leukocytes at both day 30 (*P* < 0.05) and day 60 (*P* < 0.001) of Mtb infection compared to naïve guinea pigs. In the spleen, CD1b expression was increased at all three infection endpoints compared to time-matched naïve controls (*P* < 0.05 – *P* < 0.01).

### CD1b orthologs have distinct spatial cellular expression profiles

**Fig 2:**
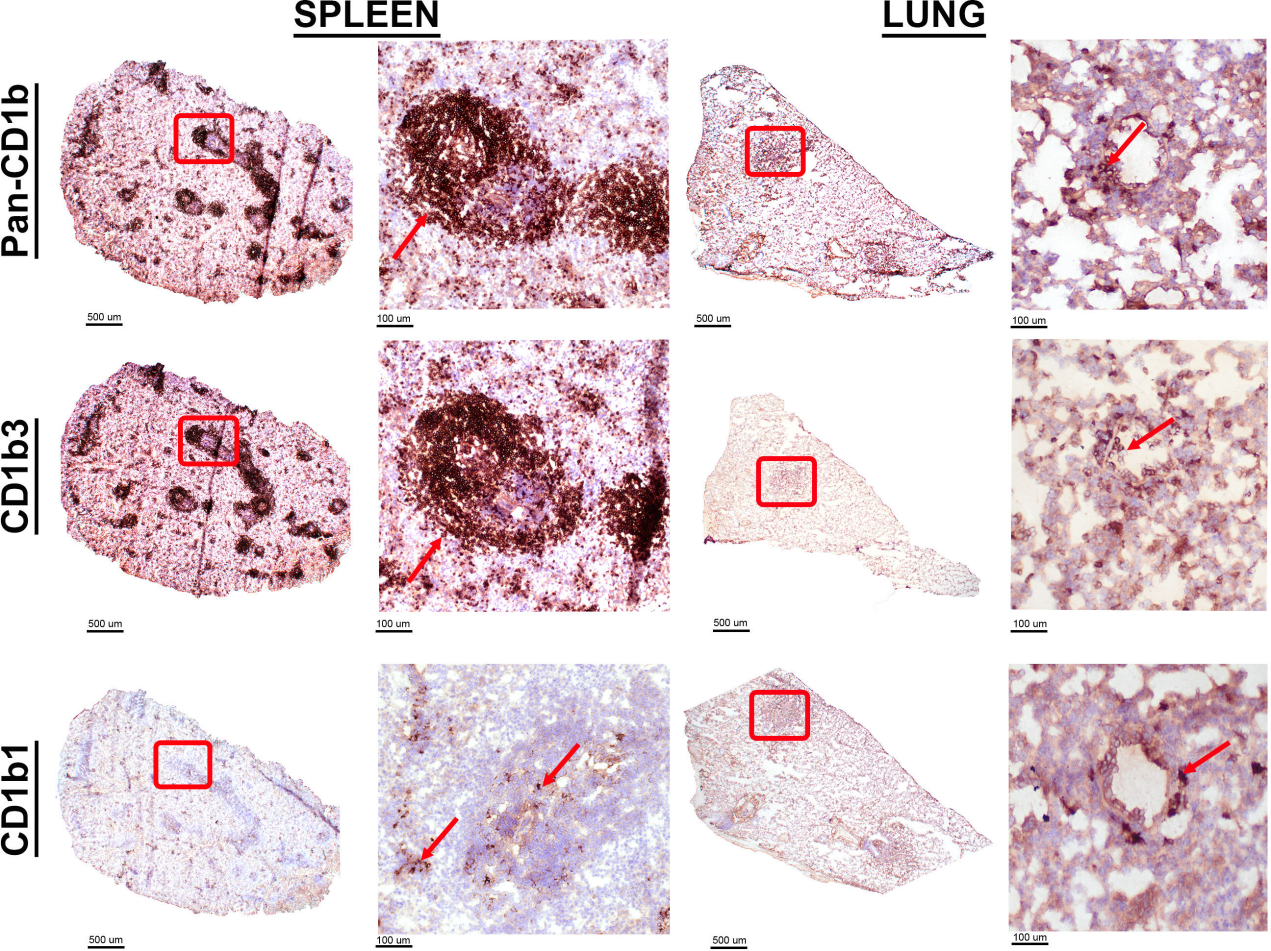
Tissue distribution of specific CD1b orthologs. Fresh-frozen naïve lung and spleen tissue cryosections were fixed in absolute methanol and labeled by immunohistochemistry. Distribution of all CD1b orthologs was detected with pan anti-CD1b antibody, 6B5. Distribution of the CD1b3 ortholog was detected with the anti-CD1b3 monoclonal antibody, MsGP9. Distribution of the CD1b1 ortholog was detected with anti-CD1b1 monoclonal antibody, 1D12. High-magnification images are representative of the areas delineated by red boxes in each subgross image. Red arrows point to cells immunolabeled by each CD1-targeting monoclonal antibody.

Having identified changes in CD1b protein expression among leukocytes responding to Mtb infection by flow cytometry in the lung and spleen, we next sought to evaluate the expression of specific CD1b orthologs. The monoclonal antibodies, 1D12 and MsGp9, are specific for CD1b1 and CD1b3, respectively (Fig. S1). Lung and spleen tissue from naïve guinea pigs were evaluated for expression of these specific orthologs by immunohistochemistry on frozen tissue (Fig. 2). Cell- and ortholog-specific expression patterns were evident across lung and spleen tissue. In the lung, alveolar and interepithelial cells within the airways were identified with 1D12, which are anatomically and morphologically consistent with alveolar and interepithelial macrophages. By contrast, MsGp9 stained the B-cell regions of the splenic white pulp, corroborating previous literature (20). In contrast to the MsGP9 antibody staining, detection of CD1b1 in the spleen using the 1D12 antibody is rare and limited predominantly to cells within the sinuses of the splenic red pulp.

**Fig 3:**
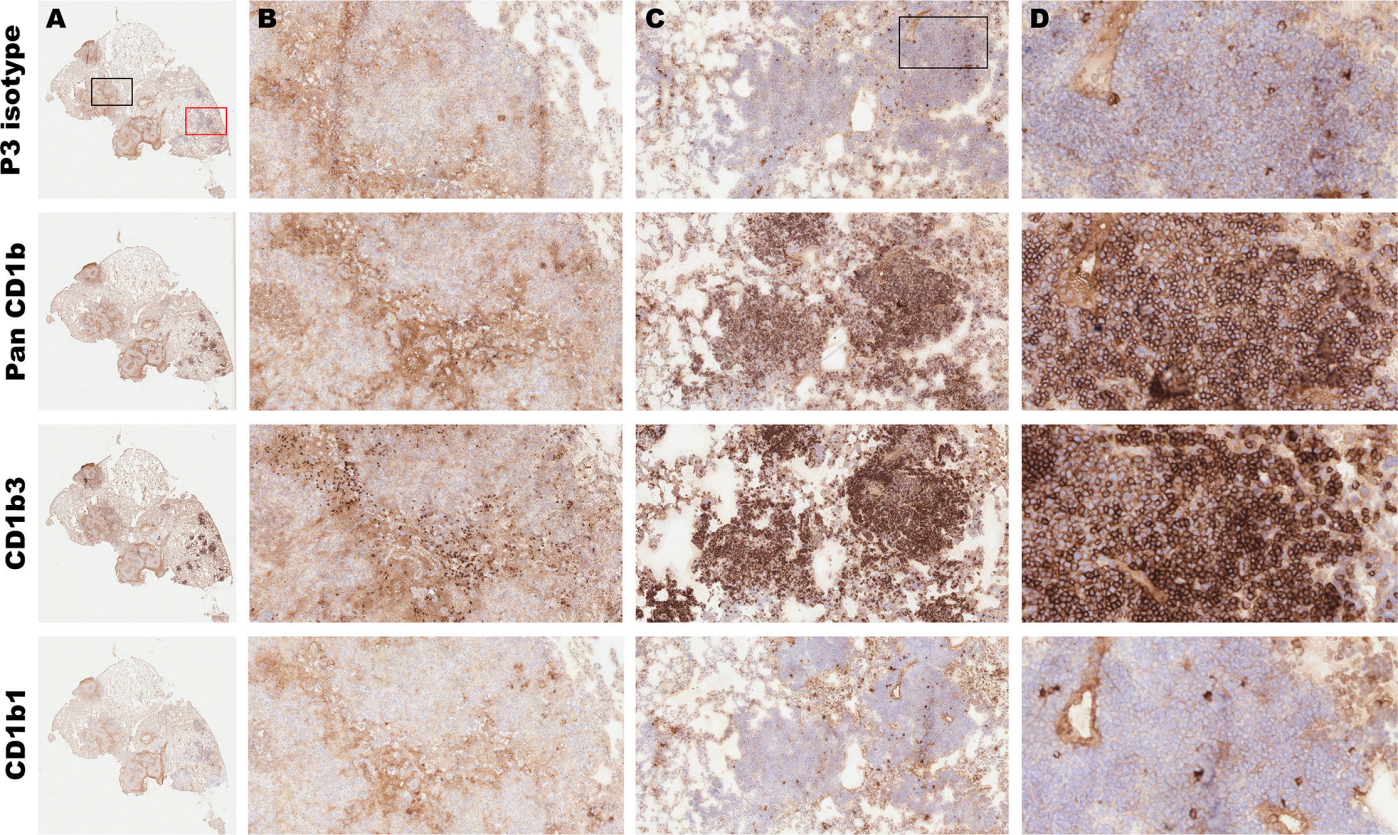
Cellular distribution of CD1b expression in the pulmonary granuloma. Fresh frozen serial sections from the lung of a Mtb-infected guinea pig at 60 days post-infection were fixed in absolute methanol and labeled by immunohistochemistry using monoclonal antibodies against all CD1b orthologs, or specifically targeting CD1b1 and CD1b3, and compared to an isotype control. (A) Low magnification demonstrates staining patterns with each antibody where a representative granuloma region is outlined in black and de novo tertiary lymphoid inflammation is outlined in red. 6× magnification (B) Representative granuloma region outlined in black demonstrating the distribution of CD1b expression. 100× magnification (C) Representative tertiary lymphoid region outlined in red demonstrating the distribution of CD1b expression. 100× magnification (D) Distribution of CD1b expression on cells within tertiary lymphoid region outlined in (C). 400× magnification.

The same chromogenic IHC approach and antibodies were utilized to evaluate CD1b ortholog spatial expression within lung granulomas. Serial sections of lung from five infected guinea pigs at the day 60 endpoint were evaluated. IHC performed with the pan-CD1b antibody, 6B5, highlights a population expressing CD1b within the lymphocytic regions at the periphery of primary granulomas and in *de novo* tertiary lymphoid structures within lung parenchyma. Further delineating ortholog-specific expression, IHC with the CD1b3-specific MsGp9 clone demonstrated strong membranous immunolabeling among morphologically small cells consistent with lymphocytes and highlights that the majority of CD1b expression in the pulmonary response manifests as the CD1b3 ortholog (Fig. 3; Fig. S7). By contrast, CD1b1 assessed by IHC with clone 1D12 showed membranous immunolabeling of few cells in the wall of the granuloma and less in tertiary follicles, as compared to the P3 isotype control (Fig. 3; Fig. S7). The clear differences across tissue and cellular distribution of CD1b1 and CD1b3 expression suggest that tissue compartments and cellular populations may have specific regulation of CD1b orthologs in naïve tissue and during the course of Mtb infection.

**Fig 4:**
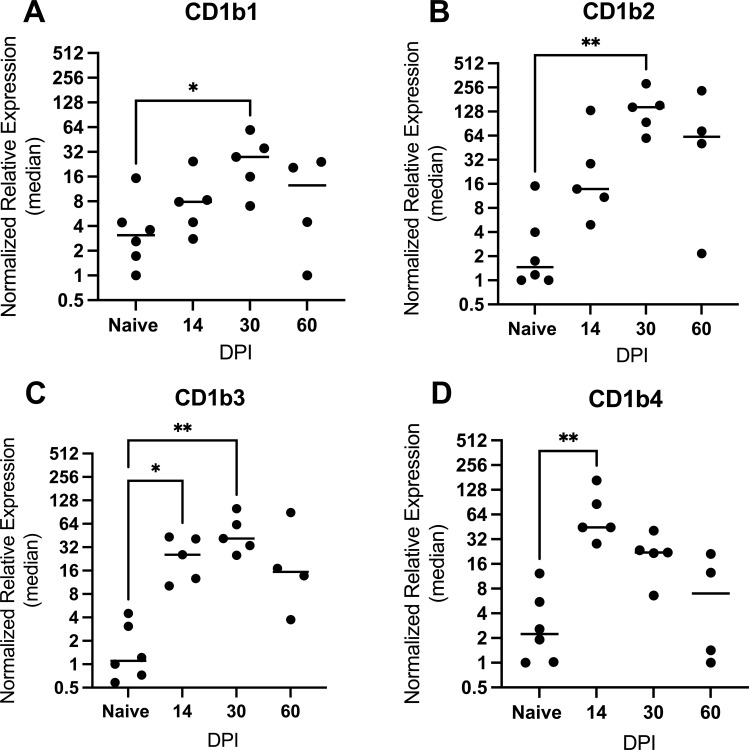
Kinetic gene expression of each CD1b ortholog at 14, 30, and 60 days post-Mtb infection. Relative gene expression of each ortholog was measured by quantitative RT-PCR on total RNA isolated from lung samples of Mtb-infected and naïve uninfected guinea pigs. Expression was scaled to the naïve control animal having the lowest expression of the CD1b1 ortholog, designated at a relative expression of 1. *P ≤ 0.05; **P ≤ 0.01; One-way ANOVA multiple comparison with a Dunnet correction.

Prior studies in humans indicated that CD1b upregulation is mediated mainly through transcriptional control (15). As a second approach to dissect how specific CD1b orthologs are regulated during the course of Mtb infection, we designed ortholog-specific primers targeting CD1b1-b4 and evaluated kinetic changes in relative gene expression in total RNA isolated from infected and naïve lung. The relative gene expression of CD1b orthologs (CD1b1-b4) performed on total lung cDNA demonstrated altered expression of CD1b in infected guinea pigs compared to total lung cDNA from naïve guinea pigs (Fig. 4). Collectively, all CD1b orthologs increased expression over the course of Mtb infection, with peak expression for CD1b1-3 occurring at day 30 of infection. CD1b2 showed the greatest increase in relative expression during infection, beginning at day 14 and reaching a peak at day 30 with a 36-fold mean increase (147.3 ± 86.1; *P* ≤ 0.01) compared to naïve controls. By contrast, CD1b4 peaked early at day 14 of infection with an 18-fold mean increase (73.9 ± 55.84; *P* ≤ 0.01) then progressively waned over days 30 and 60 of infection. Thus, the timing of CD1b transcriptional induction and waning is similar across CD1b orthologs in the pulmonary response to Mtb infection, although antibody-based detection shows expression of different cell types.

### CD1b expression patterns on lymphocytes in granulomas

**Fig 5:**
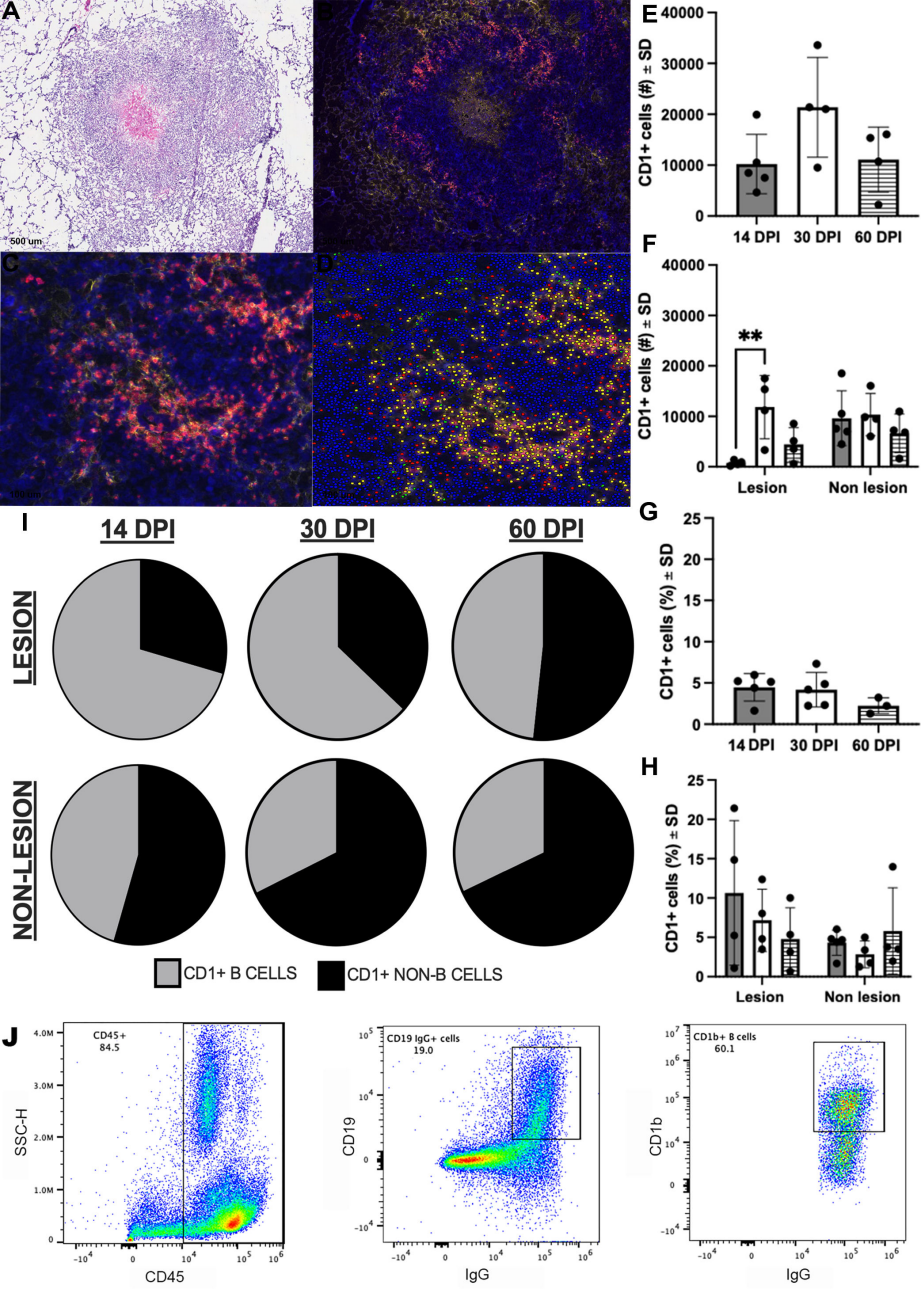
Location and cellular distribution of CD1b expression over the course of Mtb infection. Fresh frozen sections of the lung and spleen were fixed in absolute methanol and labeled with antibodies targeting all CD1b orthologs (6B5, Opal 520) or the B-cell transcription factor, Pax5 (clone 24, Opal 690), using Opal fluorochrome immunohistochemistry and nuclear counterstain, DAPI. (**A–D**) Representative sections of a lung granuloma demonstrating H&E-stained image of the granuloma labeled by IHC (**A**); Multiplexed IHC detecting CD1b (yellow) and Pax5 (red) demonstrating the distribution of expression across the granuloma (**B**); high-magnification view of CD1b+, Pax5+ region designated by the red boxes in panels A and B (**C**); and the same high-magnification field of view overlayed with Visiopharm analysis annotation of CD1b+ and Pax5+ B cells (yellow), CD1b- and Pax5+ B cells (red), or CD1b+ and Pax5- representing CD1b+ non-B cells (green) (**D**). (**E and F**) Quantitative output of CD1b+ cell numbers achieved with Visiopharm image analysis across the entire lung section (**E**) or separated into lesional and non-lesional regions of each lung section at 14 (gray bar), 30 (white bar), and 60 (hashed bar) days post-infection (DPI) (**F**). (**G and H**) The proportion of CD1+ cells among all detected cells in the whole lung (**G**) or separated into lesional and non-lesional regions of each lung section at 14 (gray bar), 30 (white bar), and 60 (hashed bar) days post-infection (**H**). (**I**) Proportional distribution of CD1+ cell phenotypes as pie diagrams across lesional and non-lesional lung tissue. (**J**) Confirmation of CD1b expression on B cells by flow cytometry. Leukocytes in spleen cell suspension were gated by expression of CD45. Among CD45+ cells, B cells were identified by co-expression of CD19 and surface IgG, then gated on CD1b expression. **P < 0.01; one-way ANOVA with a Tukey correction.

In humans, all four CD1 proteins are expressed on thymocytes, CD1c and CD1d are expressed on B lymphocytes, and CD1b expression is usually restricted to myeloid cells (15). Our data describing CD1b expression on guinea pig cells demonstrated two tissue patterns with apparent expression of CD1b on large and small leukocytes, which are likely to be myeloid and lymphoid cells, respectively, based on histological morphology and flow scatter parameters. This is divergent from what is described in the human system. We further pursued CD1b expression *via* multiplex IHC to identify the cell phenotypes and locations of CD1b-expressing leukocytes within infected lung parenchyma (Table S6). Multiplexed immunohistochemistry showed the majority of CD1b expression colocalizes with Pax5+ cells located along the periphery of Mtb granulomas, which is phenotypically consistent with B cells (Fig. 5A through D). Correlating with flow cytometric analyses, CD1b was kinetically regulated during the course of Mtb infection, with the highest number of CD1b expressing cells appreciated at 30 days post-infection, with a mean of 21,369 ± 9,829 CD1b+ cells per animal among lung granulomas (Fig. 5E and F).

Whereas prior studies of mycobacterial modulation of human CD1b expression were mainly conducted on pure cells exposed to mycobacteria or mycobacterial products *in vitro*, these *in vivo* studies provided a window into evolving *in vivo* responses. The concordance of transcripts and protein kinetics was consistent with the transcriptional regulation of CD1b proteins. Interestingly, the frequency of CD1b + cells in TB granuloma lesions reflects a large proportion of the initial cells recruited in the early response to Mtb infection, as evidenced by the increased proportion of CD1b+ cells among all granuloma-associated infiltrate at day 14 of infection (Fig. 5G and H). As such, these data fulfill predictions that mycobacterial infection induces CD1b needed for the presentation of mycobacterial lipids (41) and are consistent with a natural role of CD1b in host response. Because these cells largely express Pax5 (Fig. 5I), a master regulator of transcription driving B-cell differentiation (42), this represents a population of early responding B cells that may represent a gateway to subsequent lipid-restricted immunity. Collectively, evaluation of CD1b expression by IHC methods demonstrates that Pax5+ populations dominate among CD1b−expressing cells in the granuloma, with a paucity of CD1b expression among leukocytes in the walls of TB granulomas that lack expression of the Pax5 marker. To build further evidence of CD1b expression among B-cell populations, we performed flow cytometry on naïve guinea pig splenocytes to evaluate CD1b expression among viable CD45+ leukocytes that express CD19 and surface IgG, markers that also define B cells. Among CD45+ leukocytes, 19% expressed both CD19 and IgG. Among the CD19+ and IgG+ cells, 60.1% expressed CD1b (Fig. 5J). These data correspond with the anatomic localization of Pax5 expression among splenic B cells and colocalization with CD1b expression (Fig. S8). This further identifies B cells as a major leukocyte population expressing CD1b.

### Infection elicits CD1b-restricted cytotoxicity

**Fig 6:**
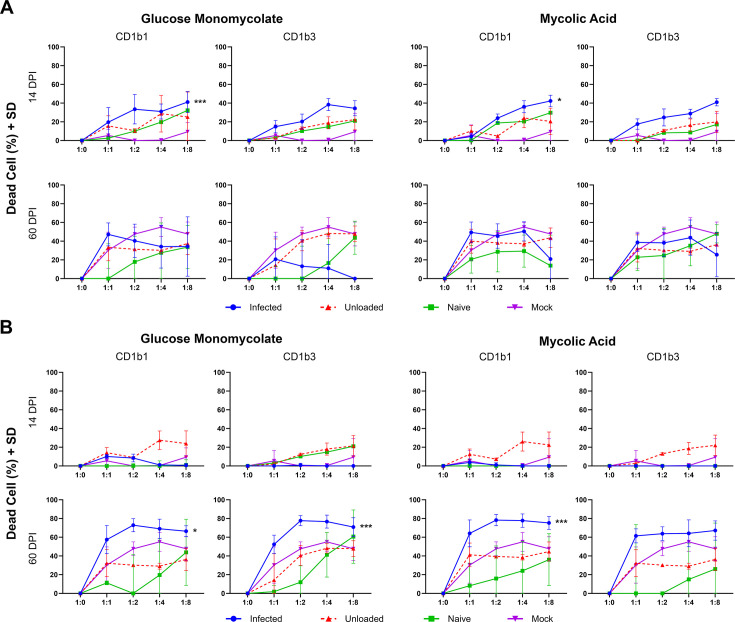
Lipid antigen-specific and CD1b-restricted cytotoxic activity among cells isolated from Mtb-infected lung and spleen. Guinea pig 104C1 fibroblasts transfected with the empty vector (Mock) or with CD1b1 or CD1b3 were labeled with CellTrace Violet (CTV) tracking dye then mock-loaded (unloaded) or loaded with synthetic glucose monomycolate or mycolic acid Mtb lipids to be used as target cells in this cytotoxicity assay. Target cell fibroblasts were incubated with non-adherent cells isolated from the lung or spleen of Mtb-infected guinea pigs at indicated ratios for each infection endpoint. Fibroblasts in co-culture were identified by flow cytometric gating on CTV-labeled cells and viability was assessed using permeability dye exclusion. (**A**) Fibroblasts incubated with effector cells derived from the lung at day 14 or day 60 of infection. (**B**) Fibroblasts incubated with effector cells derived from the spleen at day 14 or day 60 of infection. *P < 0.05, **P < 0.01, and ***P < 0.001; comparing infected to both unloaded and mock controls by one-way ANOVA with Tukey post hoc correction for multiple comparisons.

To examine the functional response of CD1b-restricted immunity to Mtb lipids over the course of infection, non-adherent effector cells isolated from spleen and lung parenchyma were incubated with CD1b1- and CD1b3-transfected fibroblast cell lines loaded with synthetic lipid antigens, mycolic acid, or glucose monomycolate. Evidence of antigen-specific cytotoxicity was defined as a statistically significant increase in cell death among lipid antigen-loaded CD1b expressing transfectants above the basal level of cell death measured among both controls, including CD1b transfectants without lipid antigen and transfectants that do not express CD1b. Based on this definition, lung effector cells (Fig. 6A) demonstrated significant antigen-specific cytotoxicity at day 14 of infection specific to the CD1b1 ortholog loaded with both glucose monomycolate and mycolic acid (*P* < 0.001 and *P* < 0.05, respectively). Cytotoxicity was not observed in the lung at later endpoints of TB disease, nor was it found against the CD1b3 ortholog loaded with lipid antigens. At day 60 of infection, corresponding to the point of greatest CD1b expression, specific cytotoxicity was observed in spleen tissue against CD1b1 loaded with either glucose monomycolate (*P* < 0.05) or mycolic acid (*P* < 0.001) and against CD1b3 loaded with glucose monomycolate (*P* < 0.01). The highest proportion of cytotoxicity was appreciated at a 1:2 target-to-effector cell ratio and maintained through ratios of 1:4 and 1:8. By contrast, naïve guinea pigs never demonstrated significant cytotoxicity above control conditions in any organ or endpoint evaluated. Significant cytotoxicity was not observed at 30 days post-Mtb infection (Fig. S9). Altogether, these data indicate that the induction of CD1b in Mtb infection corresponds to the establishment of CD1b lipid-antigen-restricted cytotoxic activity.

### CD1b-restricted immunity is not detected among PBMCs

We assessed kinetic CD1b expression and CD1b-restricted cytotoxic capacity among PBMCs to determine how CD1b-restricted immunity is manifested over the course of Mtb infection in peripherally accessible samples. PBMCs, collected *via* weekly antemortem blood draws and from our specified terminal endpoints, were analyzed for CD1b expression and CD1b-restricted cytotoxicity. Using the 1B12 monoclonal antibody, we failed to detect significant upregulation in the PBMC populations by flow cytometry across any of the specified collection points over the course of Mtb infection, or when compared to specified time-matched naïve guinea pigs (Fig. S10). Due to the requirement for high cell numbers, functional cytotoxicity assays were performed only on PBMCs collected at the specified termination endpoints. Regardless of the infection endpoint, CD1b ortholog-expressing target cell, or Mtb lipid antigen, no cytotoxic effect was demonstrated among PBMCs isolated from infected guinea pigs compared to PBMCs from naïve guinea pigs (Fig. S10).

### Mtb lipid antigens fail to elicit an intradermal response

Testing of intradermal T-cell response to purified protein derivative (PPD) is a mainstay of TB diagnosis that relies on antigen-specific recall T-cell responses. To assess whether pure, synthetic Mtb lipid antigens alone could elicit an immunologic recall response during Mtb infection, infected and naïve guinea pigs were injected with synthetic glucose monomycolate or mycolic acid lipids in either a PBS or liposomal formulation. Regardless of the formulation administered, synthetic lipid antigens delivered at 14, 30, or 60 days of infection failed to elicit a detectable delayed-type hypersensitivity response to pure antigens. By contrast, and as expected, Mtb PPD elicited a dermal response at day 30 and day 60 of infection, matching the expected time for early and late adaptive phases of T-cell response, with no antigen-specific response to PPD identified at day 14 of infection (Fig. S11).

### Enumeration of tissue bacterial burden

To confirm infection outcomes consistent with previously reported guinea pig Mtb infection studies, the burden of Mtb was assessed *via* culture of lung homogenates and the formation of colony forming units (CFU). CFU counts were consistent with validated and published low-dose aerosol exposure in the guinea pig model (Fig. S12) (27). The mean CFUs per gram of tissue were 4.56 ± 0.30, 4.79 ± 0.45, and 4.76 ± 0.13, for days 14, 30, and 60 of infection, respectively.

## DISCUSSION

There exists clear evidence for clonal (2, 11, 43) and polyclonal responses of human T cells to mycobacterial mycolate antigens (10) across human TB patient populations (26). However, major questions remain regarding the timing and location of CD1-mediated T-cell responses during Mtb infection that can be resolved with a tractable *in vivo* model. With regard to timing, CD1-mediated T-cell responses might be rapid and innate in nature, like those of NKT cells. However, there is currently limited evidence for pre-expansion of CD1b-reactive T cells, so T-cell responses might require T-cell expansion, like acquired MHC-restricted responses that develop over 3 weeks or longer. A third hybrid model is that Mtb rapidly sheds TLR agonists and other signals to induce group 1 CD1 locally at the site of infection so that CD1 protein expression is linked in time and location to shed bacterial lipid antigens (41). This latter concept is supported by *in vitro* studies showing the upregulation of group 1 CD1 transcripts and proteins within 24 to 72 hours of infection, bacterial exposure, or TLR agonist exposure (15, 44). Other studies show loss of CD1 expression on human myeloid cells *in vitro*, which generally occurs at time frames greater than 6 days when myeloid cell death ensues (45, 46). Currently, specific mechanisms of downregulation are unknown.

Our *in vivo* model begins to dissect the timing and nature of altered CD1b expression during experimental low-dose pulmonary Mtb infection. We found clear evidence for local upregulation of CD1b proteins and all four guinea pig CD1b orthologs at the transcriptional level. Moreover, we demonstrate local CD1-restricted cytotoxicity targeting two group 1 CD1b proteins, CD1b1 and CD1b3, which is evident in the pulmonary response at day 14 of infection and established disease at day 60 of infection in the spleen. We further provide *in vivo* evidence for increased transcription occurring prior to the generation of lipid antigen-specific responses as measured by antigen-restricted cytotoxicity, all of which support the hybrid model and the likely need for weeks of T-cell expansion *in vivo* after exposure to the pathogen. The identification of CD1b induction by day 14 of infection may suggest a role for this induction in the early pulmonary response to Mtb infection. These data support and extend prior *in vitro* work in human systems that observed increases in CD1b expression, where the linkage of the timing and extent of change in transcripts and proteins is consistent with transcriptional upregulation. Furthermore, our *in vivo* kinetic data match findings from one human study showing upregulated CD1b protein expression in Mtb-infected human lungs with regard to timing, although expression on specific cell types may differ (47).

Considering the location of T-cell response *in vivo*, CD1b-restricted cytotoxic activity was demonstrable in infected lung and spleen tissue, but not in the periphery. Our early efforts to study intradermal responses to lipids may have been limited by a lack of adjuvants or other formulation issues. However, it is notable that *in vivo* guinea pig CD1b system outcomes seen here are different from previously reported MHC-restricted T cells in the sense that we could not detect recall responses in the peripheral blood or skin. Thus, CD1b-restricted responses may be more localized to the site of infection as compared to the systemic nature of MHC-restricted responses. This outcome stands in contrast to a previous study in BCG-immunized guinea pigs where recall responses to skin were demonstrated in response to glucose monomycolate in the absence of Mtb infection (48), potentially explained by the purification process of the antigen rather than the use of synthetic antigens, intradermal immunization rather than pulmonary infection, or differences between BCG and Mtb organisms.

It is notable that cytotoxicity was identified as a major readout of localized functional activity of CD1b-restricted T cells, a feature that is also reported in human CD1-restricted T cells isolated from peripheral blood (49). We observed cytotoxic activity in an infection-dependent and antigen-restricted manner. Synthetic lipid antigens, mycolic acid and glucose monomycolate, both served as antigen targets for the induction of cytotoxic activity, which was observed to a greater degree in antigen-loaded CD1b1 or CD1b3 transfected fibroblast target cells exposed to splenic effector cells isolated at day 60 of Mtb infection. Synthetic lipid antigens provide a significant strength as they lack contaminating protein antigens, which may be present in lipid antigens purified directly from the Mtb organism. This approach eliminates the possibility that the antigen-restricted functional response observed in this study is the result of contaminating protein antigens. Altogether, this study provides evidence that CD1-restricted killing of target cells in active TB requires a lipid antigen in association with CD1.

One unexpected difference between the human and guinea pig CD1b systems is that we have identified that CD1b expression is primarily localized to B cells within granulomatous lesions of the spleen and lung, suggesting this cell population may contribute a previously unrecognized role to the development and maintenance of CD1-restricted immunity. This outcome is different from the human system, where CD1c and CD1d are expressed on B cells, and CD1b is not (50, 51). It was noted that the predominant lesion-associated CD1-expressing cell is the Pax5+ B cell. Although many Pax5+ B cells express CD1b, there is also a substantial population of Pax5+ B cells that are negative for CD1b in granuloma lesions. The difference in function between these cells and their contribution to ongoing granuloma-associated immune responses remains unexplored. We also observed that there is a proportionally greater frequency of CD1+ and Pax5+ B cells in the early immune response at day 14 of infection. This early manifestation of CD1 expression might suggest an efficient and early presentation of lipid antigens and an unexpected role for B cells in the early response to Mtb infection. Of note, there is a paucity of CD1b expression among non-B cell phenotypes within the granuloma, suggesting that granuloma-associated macrophages do not express CD1b orthologs. The cause and consequence of this apparently absent expression among lesion-associated myeloid cells remains unknown. The antibodies used in this study are specific for CD1b; thus, we cannot rule out a different cellular distribution expression profile of CD1c orthologs.

We also observed that CD1b1 and CD1b3 have remarkably different spatial cellular expression patterns in naïve tissues of interest. The presence of CD1b1 on alveolar macrophages in the lung, as detected by IHC among cells localized within alveolar spaces, and macrophage-like cells in the splenic red pulp would suggest that CD1b1 is a myeloid-cell-associated CD1b ortholog. Additional surface marker reagents are needed to better assign myeloid cell-specific expression. By contrast, CD1b3 was highly expressed within the B-cell zone of the splenic white pulp, which can be discretely identified by the expression of Pax5 as a highly specific marker of B-cell populations. This finding indicates that CD1b3 is a B-cell-associated molecule expressed in both naïve and infected conditions. Collectively, these data suggest that CD1b orthologs may serve multiple roles in the contribution to immunity against Mtb infection.

Having now established the presence of CD1-restricted immunity at the primary sites of Mtb infection achieved by low-dose aerosol exposure in the guinea pig model, it remains critical to better understand the contributions of this branch of immunity to TB disease outcome. It is still unknown whether CD1-restricted responses provide a benefit or detriment to the host, and how these antigen presentation molecules contribute to the mix of a significantly larger repertoire of protein-based antigens in the context of classical MHC class I or class II antigen presentation.

## SUPPLEMENTAL MATERIAL

Supplemental material iai.00380-24-s0001.pdf Fig. S1 to S12; Table S1 to S6.

ASM does not own the copyrights to Supplemental Material that may be linked to, or accessed through, an article. The authors have granted ASM a non-exclusive, world-wide license to publish the Supplemental Material files. Please contact the corresponding author directly for reuse.
