# Supplementary material for: Establishment of CD1b-restricted immunity to lipid antigens in the pulmonary response to Mycobacterium tuberculosis infection
Source: Infect Immun. 2024 Nov 4;92(12):e00380-24. doi: 10.1128/iai.00380-24 (PMC11629625; doi:10.1128/iai.00380-24)
Supplement: Supplemental material — Fig. S1 to S12; Table S1 to S6. [file iai.00380-24-s0001.pdf]

Online Data Supplement

Establishment of CD1b-restricted immunity to lipid antigens in the pulmonary response to *Mycobacterium tuberculosis* infection

Macallister C. Harris, Hadley E. Gary, Sarah K. Cooper, David F. Ackart, James E. Dilisio, Randall J Basaraba, Tan-Yun Cheng, Ildiko van Rhijn, D. Branch Moody, Brendan K. Podell

TABLE OF CONTENTS

SUPPLEMENTAL FIGURES & TABLES .... PAGES

Figure S1. Anti-CD1 ANTIBODY SPECIFICITY……………………………………………….…2
Table S1. DESIGNATED SPECIFICITY OF ANTI-GUINEA PIG CD1 MONOCLONAL ANTIBODIES…………………………………………………………………………….…...3
Figure S2. CONFIRMATION OF LIPOSOME SIZE AND CELLULAR UPTAKE……………..……3
Figure S3. GATING STRATEGY FOR EVALUATING CD1B EXPRESSION BY FLOW CYTOMETRY……………………………………………………………………...………….3
Table S2. ANTIBODY AND FLUOROPHORE CONJUGATES UTILIZED IN FLOW CYTOMETRY…………………………...…………………………………………………….4
Figure S4. CD1B-RESTRICTED CYTOTOXICITY GATING STRATEGY………………………..4
Table S3. CD1B ORTHOLOG SPECIFIC PRIMER SEQUENCES………………....………...….5
Figure S5. CD1B ORTHOLOG SPECIFIC GENE BLOCK SEQUENCES…………..……………6
Figure S6. CD1B ORTHOLOG PRIMER SPECIFICITY………………….......................….7
Table S4. CHROMOGENIC IHC PROTOCOL…………………………………………...……..8
Table S5. MULTIPLEX FLUORESCENCE IHC PROTOCOL……………………….…...……..8
Figure S7. CD1B ORTHOLOG EXPRESSION IN MTB INFECTED LUNGS…………..……….9
Table S6. FREQUENCY OF CD1B EXPRESSION BY MULTIPLEX IHC...…………….……....9
Figure S8. SPECIFICITY OF PAX5 DETECTION BY IHC IN GUINEA PIG LYMPHOID TISSUE 10
Figure S9. CD1B RESTRICTED CYTOTOXICITY AT DAY 30 OF INFECTION.…………...……11
Figure S10. CD1B EXPRESSION AND CYTOTOXICITY IN PBMC SAMPLES…………………12
Figure S11. INTRADERMAL MTB LIPID ANTIGEN CHALLENGE………………………………13
Figure S12. CFU BURDEN IN INFECTED LUNG ….…………………………………………13

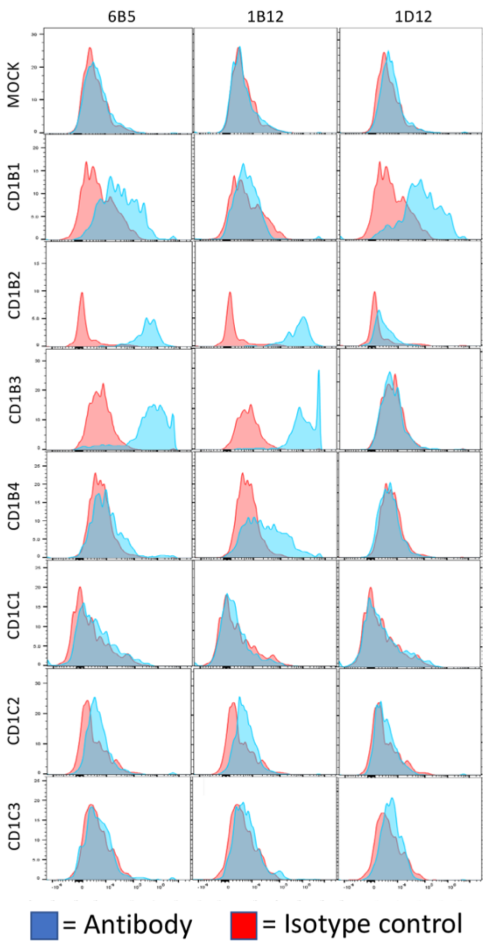

Figure S1a. CD1 ortholog specificity of monoclonal antibodies 6B5, 1B12, and 1D12. 104C1 fibroblasts transfected with specific CD1b and CD1c orthologs were labeled with each anti-CD1 antibody to determine ortholog specificity. The non-specific P3 antibody clone was used as an isotype control.

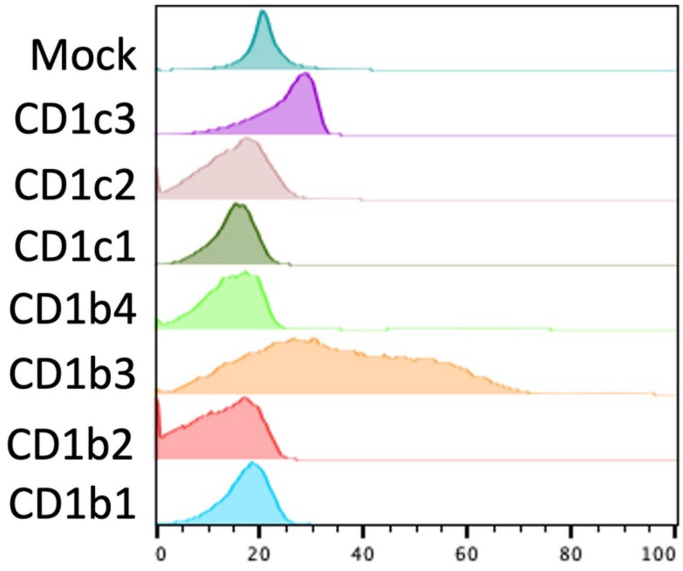

Figure S1b. CD1 ortholog specificity of the commercial monoclonal antibody, MsGp9. 104C1 fibroblasts transfected with specific CD1b and CD1c orthologs were labeled with the anti-CD1b3 antibody, MsGp9, to determine specificity for the b3 ortholog.

Table S1. Cross reactivity to specific CD1 isoforms among provided anti-CD1b/c antibodies

Anti-CD1 antibody	CD1 isoforms and orthologs detected
P3	Isotype control
1B12	CD1b2, CD1b3, CD1b4
1D12	CD1b1
6B5	CD1b1, CD1b2, CD1b3, CD1b4
BCD	CD1b2, CD1b3, CD1b3, CD1c3
MsGP9	CD1b3

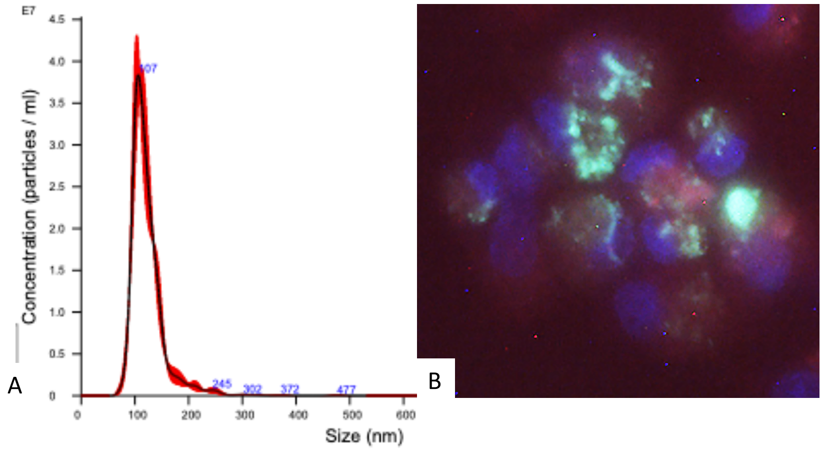

Figure S2: Liposome size and cellular uptake. A) Particle size analysis of liposomes formulated using the Avanti extrusion process. Particle size was analyzed using a Nanosight, instrument. B) Fluorescent microscopy of CellVue labelled liposomes (green) incubated with bone marrow derived macrophages (DAPI labelled) and colocalized with Lysotracker dye (red) to define acidified compartments.

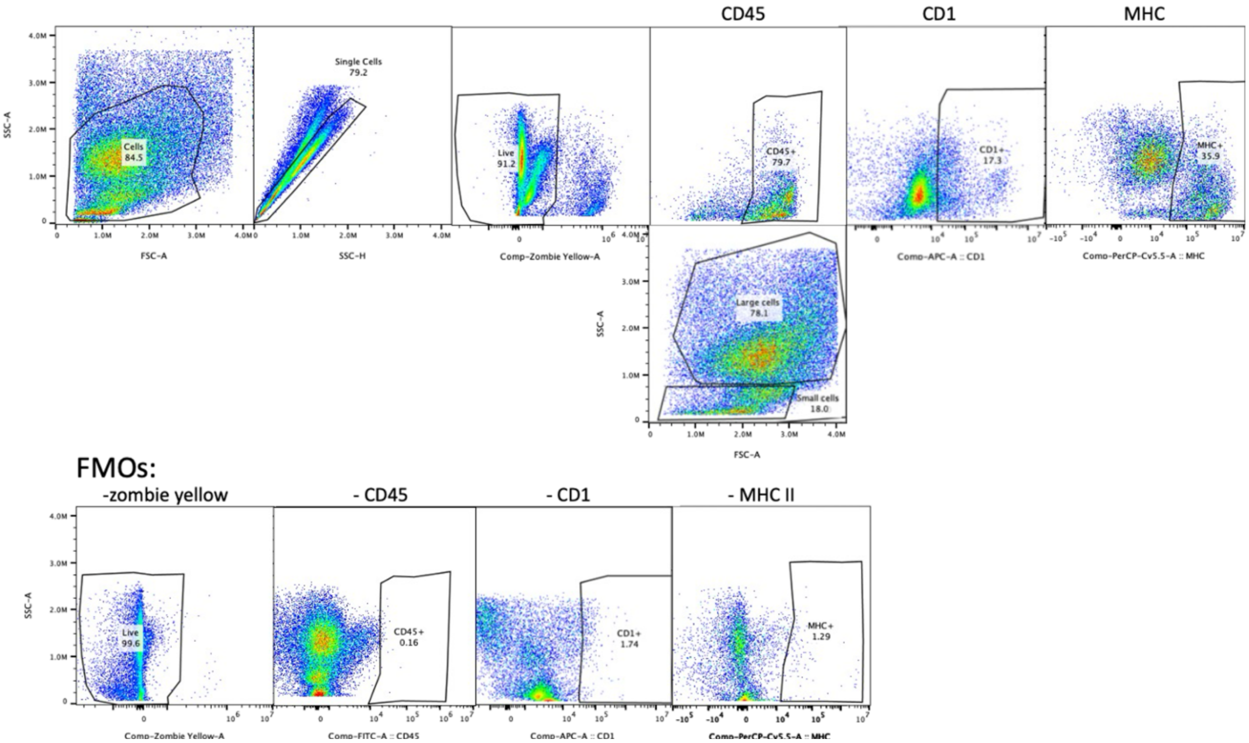

Figure S3. CD1b expression gating strategy. Initial gating on scatter parameters and single cells identifies the cell population of interest. This population is gated on viable cells only by excluding those that accumulated Zombie Yellow permeability dye. Leukocytes are the identified by CD45, evaluated in total or by large and small leukocyte scatter gating for cells expressing CD1b and/or MHC II based on FMO gating controls.

Table S2. Antibody and fluorophore conjugates used in flow cytometry assessments

Antibody Target	Fluorophore	Clone
P3	Isotype control	P3
CD45	FITC or PE	IH-1
MHC II	PE-Cy5.5	IVA12
CD11	PE	MIL4
CD1	PE-Texas Red or APC	1B12

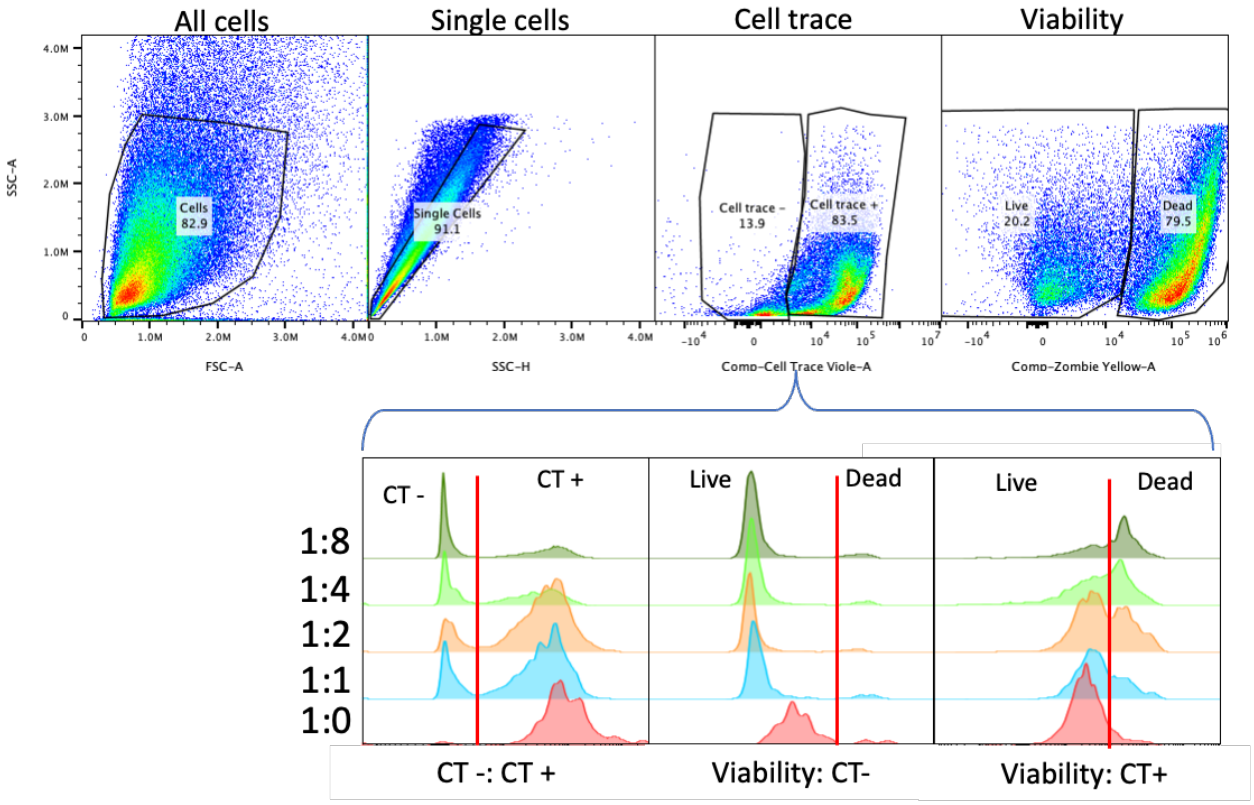

Figure S4. CD1b-restricted cytotoxicity gating strategy. Initial gating identifies the cell population of interest and single cells. Single color CellTrace Violet is used to identify guinea pig fibroblast transfectants. CellTrace Violet positive cells represent the target cells for the cytotoxicity assay and are assessed for overall viability in all samples. Histograms demonstrate the changing ratio of CellTrace Violet positive cells (target cells) to CellTrace Violet negative cells (effector cells) across multiple target to effector cell ratios as well viability among effector and target cells.

Table S3. CD1b ortholog specific primer sequences

CD1b ortholog	Primer	Sequence
**CD1b1**	Forward	5’-ACA GTG GCC AAC TTC TGT CC
**CD1b1**	Reverse	5’-ATA GTG CCT GTG TCG CTG TC
**CD1b2**	Forward	5’-GAG TTG CAT TCT GGA GGA GC
**CD1b2**	Reverse	5’-CCC AGC TTC TAG GAC ACT CA
**CD1b3**	Forward	5’-ACA AAA GCC CAC GTG AAT GC
**CD1b3**	Reverse	5’-GTC CAG TCC TCC AAA GGC TC
**CD1b4**	Forward	5’-GGC TGG TTG GGA GAT ATG GA
**CD1b4**	Reverse	5’-TTC TGG GCT TCT CTG ACG AA

CD1B1 Gene block: (PCR product: 129)

```
  1 atgctgctcg tggcactggc attgcttgca tttctcttcc ctgctggtga cactcagaat
 61 gccttacagt ggccaacttc tgtccatggc atccaaatct catccttttt caaccatacc
121 atggcacaaa gtcgatgctc aggctggttg tcccgcagat ctccaggaaa tatggagctt
181 ggtagctttg acagcgacac aggcactatc atatttaaga aaccctggtc taaagcaaac
241 ttcagcaatg aagaggtttt ggagttggag gagctatttc aagtctacat gttgggattc
301 atcagagaag tacaggaacg tatgagcgat ttccagatgg aatatccctt tgagatccag
361 ggcattgcag gctgtgaact gatttctgga ggaaccattg atttcttcct gagaggagct
421 ttagagggac tagatttctt gagtattaag aattctacat gttggcctgc cccagaaggt
481 ggcaccaagg caaaaaaatt
```

CD1B2 Gene block: (PCR product: 225)

```
  1 attttggaaa tggaggagtt atttcgactc tacttcttag gatttgttaa agaagtccag
 61 gaacttgtca gtgatttcca gctggaatat ccttttgaga tccagggcat tgcaggctgt
121 gagttgcatt ctggaggagc cattgtaagc ttcttgatgg gagctataga aggactgcat
181 ttcatgagca tcaataatta ttcatgtttg tcccgcagat ctccacctgc cccagaaggt
241 ggtaccaggg cacagaaatt ctgtgcacta atcttacagt acaaaggaat ttgtgatatt
301 gtggaaaatc tcctcacaaa agtttgcccc agatatctga tgagtgtcct agaagctggg
361 aaggcagctc tgcagaagca cgtgaagcct gaggcctggc tatcccaggg acccagcccc
421 gagcctggct atctgcagct ggtgtgccac gtctctggct tctacccaaa gcctgtgtgg
481 gtgatgtgga tgagaggtga
```

CD1B3 Gene block: (PCR product: 171)

```
  1 tgggtgaaat atcaactctc aggctggttg ggagatttgc agattcatgg catggacagt
 61 gattcaggca ctgccatatt cctgaagccc tggtctaagg gcaacttcag caatgaagaa
121 atgtctgaag tggtggagct atttcgggtc tatatcgttg cattcattcg agaaacaaaa
181 gcccacgtga atgccttgca gatggaatac tcccgcagat ctccaccttt tgagatccag
241 ggcattgcag gctgtgagct gcattctgga ggtgccattg taagcttttt gcaaggagcc
301 tttggaggac tggactttgt gagtttcaag aattcttctt gtgttcctgc cccagaaggt
361 ggcagcaggg cacagacagt gtgcaaacta cttgctccat atcatgatat cttctacact
421 gtggagaagc tgctgtatga aacctgtcct caatatctct tgggtgtcct tcaagcagga
481 aaggcagatc tacacaggca
```

CD1B4 Gene block: (PCR product: 125)

```
  1 atgctgctcc tggccctggc attttttttc ccagctggtg acactcagaa cgttttgccg
 61 gggaaaattt ctttctatgg catccaaatc tcaacgtttt tcaaccatac cgtggtagag
121 aatcgaggat caggctggtt gggagatatg gagattagca gctgggacag tgaaaaagaa
181 actatcatat ttcggaaacc ctggtctaaa tcccgcagat ctccaggcaa cttcagcaat
241 gacgagattt tggaggtgga agagatattt caagtctact tctttggatt cgtcagagaa
301 gcccagaaac atatgagtga tttccaggtg gaatacccct ttgagattca ggtcatctca
361 ggctgtgagg tgaactctca cagatccttt gattacttca tgagggtagc agtaaaggga
421 ttggatctcc tgagcatcaa gaatcattca tgttggcctg ccccagaagg tgtttctaga
481 gcacaggaaa tctggacatt
```

Segment gene Rv0733 in Mycobacterium tuberculosis H37Rv:

5'-tcccgcagatctcca

Figure S5. CD1b ortholog-specific gBlock sequences: 500 basepair GeneBlock sequences were synthesized to match each specific region of cDNA for CD1b1, b2, b3 and b4 targeted for PCR, then used for development and specificity confirmation of the primer sequences listed in Supplemental Table 3. Yellow highlighted regions represent the annealing sites for each forward and reverse primer set. Purple highlighted regions represent an inserted region of the Mtb Rv0733

gene, used to distinguish GeneBlock DNA from tissue-derived cDNA PCR product, for the purposes of distinguishing control Gene Block sequences from tissue-derived cDNA.

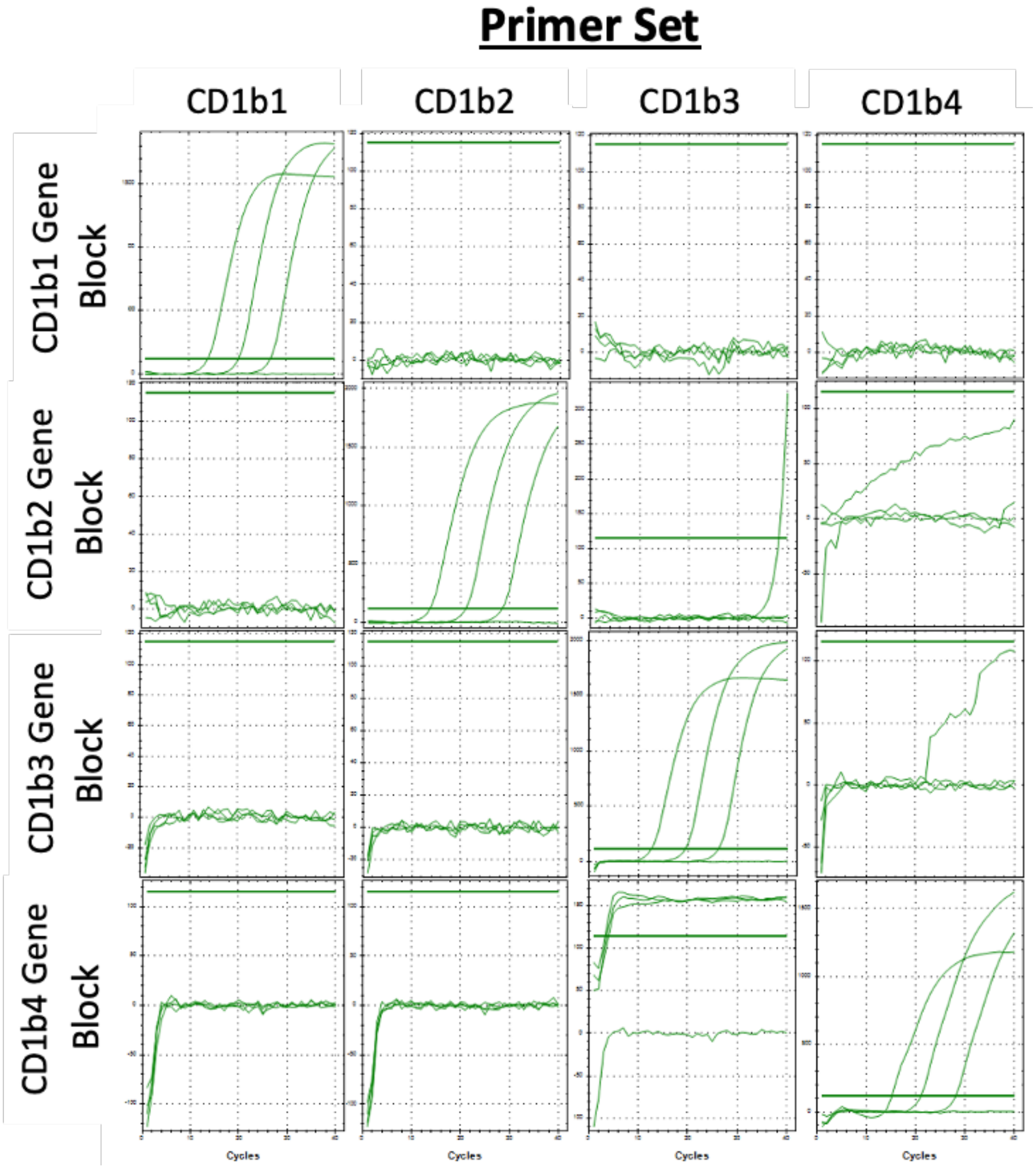

Figure S6. Confirmation of CD1b ortholog primer specificity. CD1b ortholog-specific primer pairs designated in Supplemental Table 3 were assessed for specificity against each targeted CD1b ortholog using gBlock synthetic sequences shown in Supplemental Figure 5. A checkerboard approach was used to confirm the strict specificity of each primer pair by qPCR. Standard curves of three serial 10-fold dilutions of CD1b Gene Block DNA are shown.

Table S4. Leica Bond Rx chromogenic IHC protocol

CD1 Chromogenic Protocol:

Step number	Process	Time (mins)
1	Peroxide block	10
2-8	Bond wash solution	-
9	Block (2.5% goat serum)	10
10	Primary antibody	30
11-13	Bond wash solution	-
14	Post primary HRP antibody	10
15-25	Bond wash solution	-
26	Deionized water	-
27	Polymer (Leica)	10
28-31	Bond wash solution	-
32	Deionized water	-
33	Mixed DAB refine (Leica)	0
34	Mixed DAB refine (Leica)	10
35-40	Deionized water	-
41	Bond DAB enhancer (Leica)	5
42-44	Deionized water	-
45	Hematoxylin	5
46	Deionized water	-
47	Bond wash solution	-
48	Hematoxylin DAKO A	2
49-52	Deionized water	-

+ all volumes are dispensed at 150ul per step, all steps performed at room temp

Table S5. Leica Bond Rx multiplex fluorescent IHC protocol

IHC CD1 Opal Multiplex:

Step number	Process	Time (mins)
1		
2	Kit water (Leica)	0
3-4	Bond wash solution	-
5	H202	25
6-8	Bond wash solution	-
9	Block (2.5% goat serum)	5
10	Primary antibody	30
11-13	Bond wash solution	-
14	Secondary HRP antibody	10
15-19	Bond wash solution	-
20	Opal Fluorophore 1 (Akoya)	0
21	Opal Fluorophore 1 (Akoya)	10
22-25	Bond wash solution	-
26	Bond ER 1 solution (Leica)	0
27	Bond ER 1 solution (Leica)	0*
28	Bond ER 1 solution (Leica)	20*
29	Bond ER 1 solution (Leica)	0
30-35	Bond wash solution	-
36	H202	10
37-39	Bond wash solution	-
40	Block (2.5% goat serum)	5
41	2nd primary antibody	30
42-44	Bond wash solution	-
45	Secondary HRP antibody	10
46-50	Bond wash solution	-
51	Opal Fluorophore 2 (Akoya)	0
52	Opal Fluorophore 2 (Akoya)	10
53-59	Bond wash solution	-
60	DAPI	11
61-65	Bond wash solution	-

+ all volumes are dispensed at 150ul per step

*denotes 95C temperature incubation, all other steps are completed at room temp

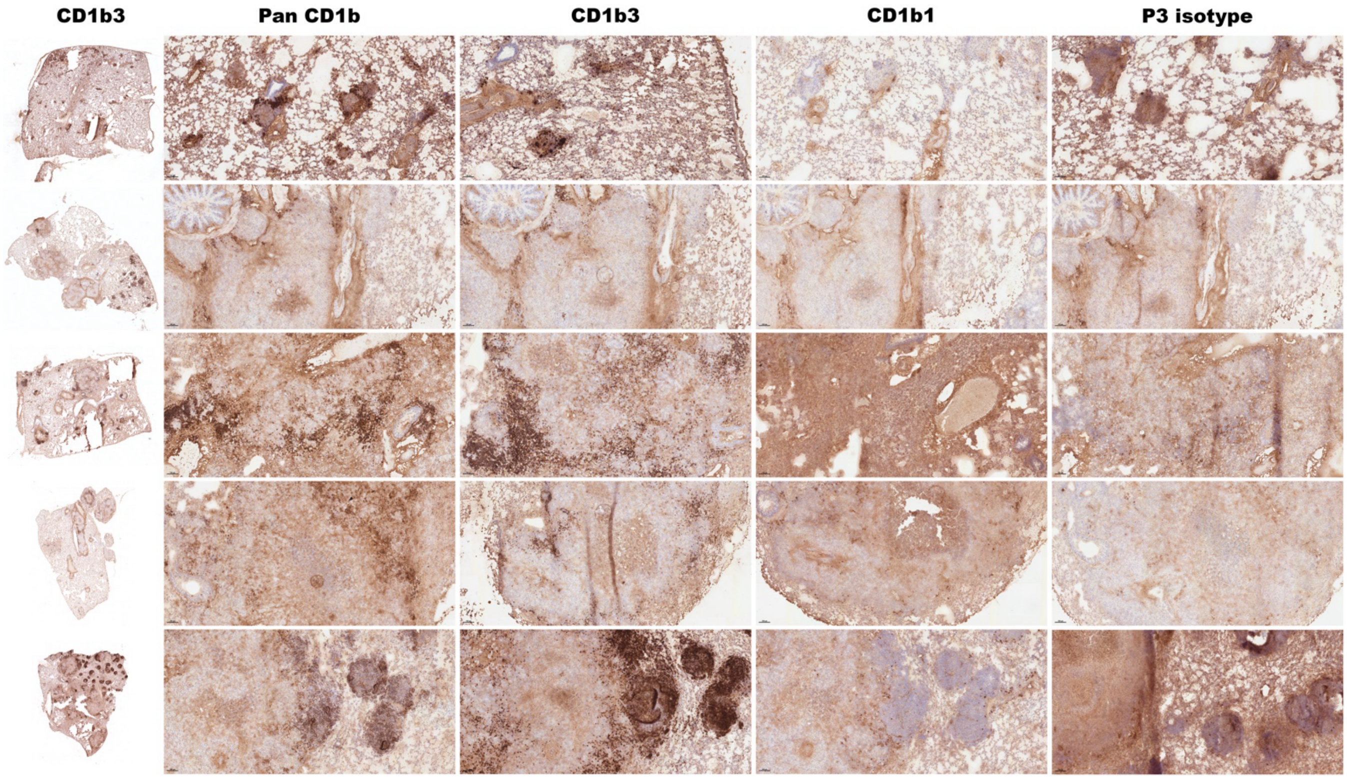

Figure S7. Expression of total CD1b orthologs, or specifically CD1b3 or CD1b1 in Mtb infected lung. Lungs collected at 60 days of Mtb infection were embedded in OCT media for frozen sectioning. IHC was performed on frozen sections from five different guinea pigs. Subgross images of lungs are shown stained with the CD1b3-specific antibody, MsGp9. Higher magnification regions are shown for pan-CD1b, CD1b3 and CD1b1 or isotype control.

Table S6. Frequency of CD1b expression on Pax5+ B cells and cells not expressing Pax5 measured by multiplex IHC.

	Whole lung nucleated cells	Whole CD1+ cells	Lesion nucleated cells	Lesion CD1+ cells
**Day 14**	334163	19931	26740	1398
	151226	7512	1669	514
	238918	10507	0	0
	162614	8484	4946	919
	283379	4644	15592	172
**Day 30**	426925	21010	90332	3302
	286845	21375	439527	11168
	915754	33581	217912	15355
	691380	21369	204117	17519
**Day 60**	172154	2205	90079	596
	353576	15364	160109	8545
	302858	10733	112656	3530
	609507	16063	194176	5121

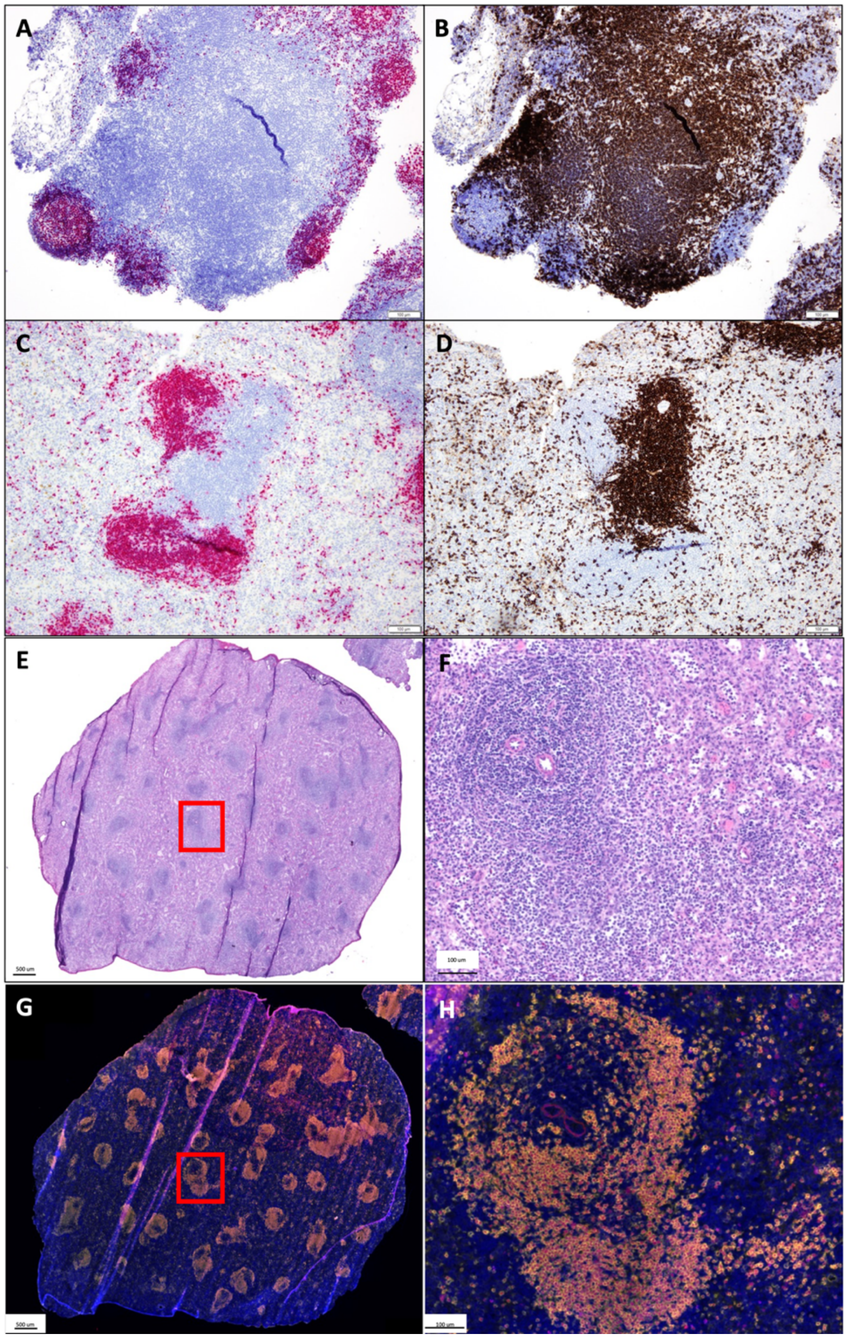
A
B
C
D
E
F
G
H
500 um
100 um
500 um
100 um

Figure S8. Specificity of Pax5 antibody clone 24 in guinea pig lymphoid tissue. Lymph node from a naïve, uninfected guinea pig shows expected orientation of (A) Pax5 expressing cells (red) within cortical follicular structures and (B) CD3-expressing cells (brown) in paracortical and medullary locations. Spleen from a naïve, uninfected guinea pig shows expected orientation of (C) Pax5 expressing cells (red) within peripheral regions of splenic white pulp and (B) CD3-expressing cells (brown) in the immediate periarteriolar lymphoid sheath of the white pulp. Fresh frozen section of spleen stained with H&E at (E) low magnification and red box designating (F) higher magnification of a white pulp follicle. A serial section of the same fresh frozen spleen labeled with dual fluorescent IHC for pan-CD1b using the 6B5 antibody (yellow) and Pax5 (red) at (G) low magnification and red box designating (H) higher magnification of dual-labeled CD1+ and Pax5+ cells.

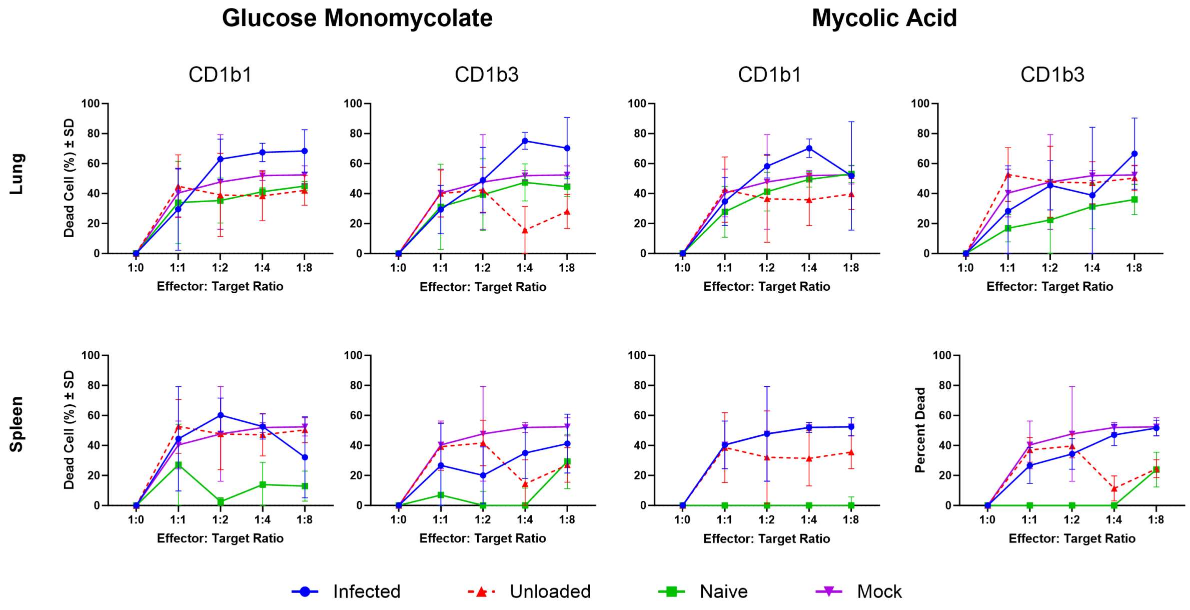

Figure S9. CD1b-restricted cytotoxic activity among cells isolated from Mtb-infected lung and spleen at 30 days post-Mtb infection. Guinea pig 104C1 fibroblasts transfected with the empty vector (Mock) or with CD1b1 or CD1b3 were labeled with CellTrace Violet (CTV) tracking dye then mock-loaded (Unloaded) or loaded with synthetic glucose monomycolate or mycolic acid Mtb lipids to be used as target cells in this cytotoxicity assay. Target cell fibroblasts were incubated with non-adherent cells isolated from the lung or spleen of Mtb-infected guinea pigs at indicated ratios for each infection endpoint. Fibroblasts in co-culture were identified by flow cytometric gating on CTV-labeled cells and viability assessed using permeability dye exclusion.

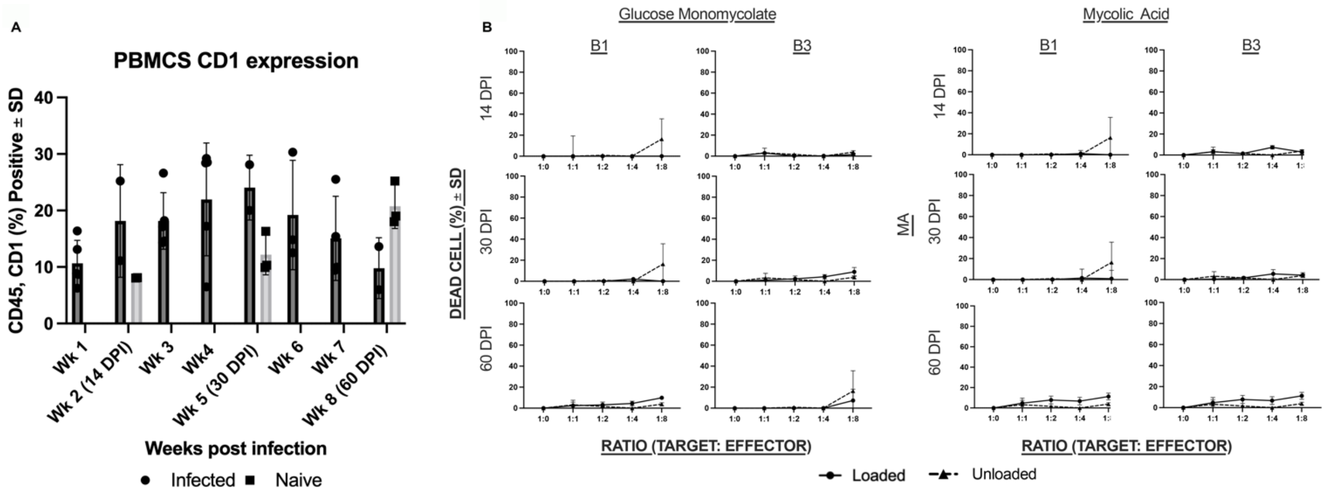

Figure S10. CD1b expression and cytotoxic activity in peripheral blood of naïve and Mtb-infected guinea pigs. (A) Cells were gated for live, CD45+ cells and evaluated for overall CD1b expression using the anti-CD1b monoclonal antibody, 1B12. (B) Guinea pig 104C1 fibroblast cells transfected with CD1b1 or CD1b3 were labeled with CellTrace Violet (CTV) tracking dye then mock-loaded (unloaded) or loaded with synthetic glucose monomycolate or mycolic acid. Target cell fibroblasts were incubated with PBMCs derived from Mtb-infected guinea pigs. Fibroblasts in co-culture were identified by flow cytometric gating on CTV-labeled cells and viability assessed using permeability dye exclusion.

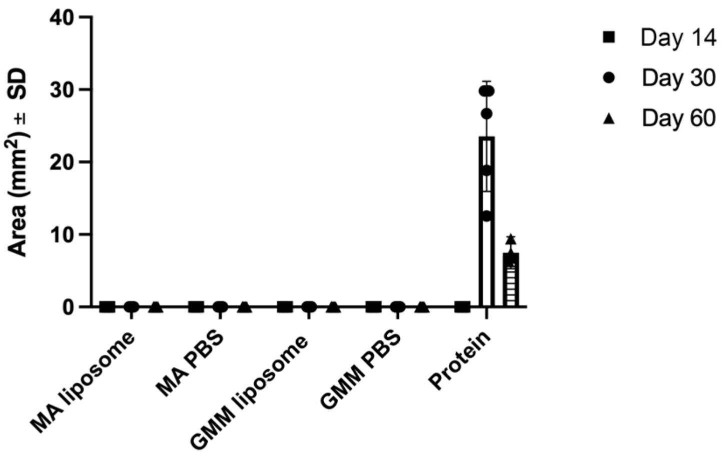

Figure S11. Intradermal Mtb lipid antigen challenge. At 48-hours prior to specific endpoint termination, Mtb-infected and naïve guinea pigs were administered intradermal injections of glucose monomycolate, mycolic acid, purified protein derivative (PPD), or saline control, either free in liquid suspension or incorporated into liposomes. Data is expressed as area of induration 48 hours after administration. Reaction was not observed at any saline negative control injection site, or among any antigen preparation across the naïve guinea pigs (data not shown).

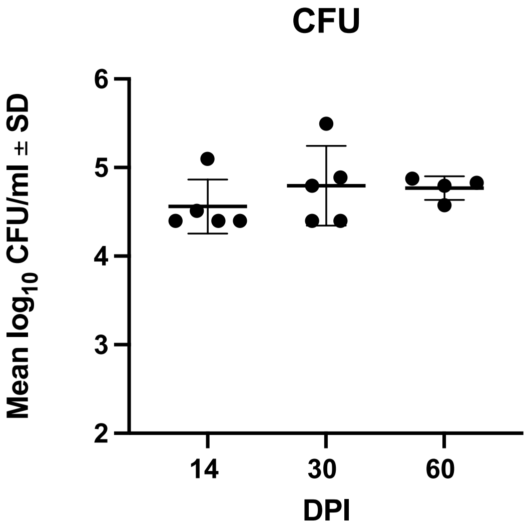

Figure S12. Mtb burden in lung at all experimental endpoints. CFU burdens derived from lung tissue at 14, 30 and 60 days post-infection. CFUs were obtained and calculated via established lab protocols and are consistent with a standard low-dose aerosol guinea pig exposure.
